# Supplementary material for: ErbB2/HER2-targeted CAR-NK cells eliminate breast cancer cells in an organoid model that recapitulates tumor progression
Source: Mol Ther. 2025 Apr 24;33(8):3559–75. doi: 10.1016/j.ymthe.2025.04.033 (PMC12461661; doi:10.1016/j.ymthe.2025.04.033)
Supplement: Supplementary file 1 [file mmc1.pdf]

YMTHE, Volume *33*

Supplemental Information

ErbB2/HER2-targeted CAR-NK cells eliminate breast cancer cells in an organoid model that recapitulates tumor progression

Jasmin Röder, Tijna Alekseeva, Anne Kiefer, Ines Kühnel, Maren Prüfer, Congcong Zhang, Malena Bodden, Sebastian Rosigkeit, Anja Waldmann, Torsten Tonn, Ernesto Bockamp, Stefan Stein, and Winfried S. Wels

Supplemental Methods

ImageJ Macro to determine organoid area based on brightfield images

Language: ImageJ Macro

```
// Select the brightfield image
selectWindow("Brightfield");
// Apply Gaussian Blur to smooth the image
run("Gaussian Blur...", "sigma=1");
// Set scale in microns
run("Set Scale...", "distance=17.0 known=100 unit=micron");
// Set automatic threshold and convert to mask
//setThreshold(137, 255);
setOption("BlackBackground", true);
run("Convert to Mask");
// Apply morphological operations to clean up the mask
run("Close-");
run("Erode");
run("Watershed");
// Analyze particles (organoid area)
run("Analyze Particles...", "size=9000-400000 show=[Count Masks] display exclude clear include overlay
add composite");
```

Image-based analysis of organoid area. The macro processes a brightfield image by applying a Gaussian blur to smoothen the image and reduce noise. The image scale is set to microns using a known distance for calibration. A threshold segments the organoids, converting the binarized image into a mask. Morphological operations, including erosion and watershed, refine the mask and separate closely connected organoids. Particle analysis measures organoid sizes within a specified range, and results are displayed.

ImageJ Macro to determine nucleus area based on Hoechst staining

Language: ImageJ Macro

```
// Select the Hoechst image
selectWindow("Hoechst");
// Set scale in microns
selectWindow("Hoechst");
run("Set Scale...", "distance=312 known=100 unit=micron");
// Enhance contrast
run("Enhance Contrast", "saturated=0.35");
// Set threshold for nuclei
setAutoThreshold("Huang dark");
run("Convert to Mask");
// Apply morphological operations to clean up the mask
run("Erode");
run("Watershed");
// Analyze particles (nuclei area)
run("Analyze Particles...", "size=20-20000 display exclude clear include overlay add");
```

Image-based analysis of nucleus area. The macro processes a Hoechst-stained image to measure nucleus size. The image scale is set to microns using a known distance for calibration. The contrast of the image is enhanced, followed by automatic thresholding to segment the nuclei, which converts the image into a mask. Morphological operations, such as erosion and watershed, are applied to refine the mask and separate closely connected nuclei. Particle measures the size of the nuclei within a specified size range, and the results are displayed.

ImageJ Macro to quantify mean fluorescent intensity of p53 staining

Language: ImageJ Macro

```
// Select the image (Composite-p53-Hoechst)
selectWindow("Composite");
// Split the channels of the composite image
run("Split Channels");
selectWindow("C1-Composite");
rename("p53");
selectWindow("C2-Composite");
rename("Hoechst");
// Adjust brightness and contrast
selectWindow("Hoechst");
setMinAndMax(95, 316);
run("8-bit");
selectWindow("p53");
setMinAndMax(152, 407);
run("8-bit");
// Apply pseudo flat field correction to Hoechst
selectWindow("Hoechst");
run("Pseudo flat field correction", "blurring=1");
// Create background mask from Hoechst channel
selectWindow("Hoechst");
run("Duplicate...", "title=Hoechst_background");
selectWindow("Hoechst_background");
run("Median...", "radius=1");
setOption("BlackBackground", true);
run("Convert to Mask");
run("Watershed");
// Analyze particles
run("Analyze Particles...", "size=80-Infinity circularity=0.50-1.00 exclude overlay add composite");
selectWindow("p53");
roiManager("Measure");
```

Image-based analysis of p53 expression. The macro is designed to process a composite image containing Hoechst and p53 channels to measure p53 expression within nuclei. The composite image is split into two separate channels, with the first channel designed for p53 and the second for Hoechst. Brightness and contrast adjustments are made for both channels, and the Hoechst channel undergoes a pseudo flat field correction to enhance uniformity. A duplicate of the Hoechst channel is created and processed with a median filter to generate a background mask. The background mask is then converted to a binary mask and refined using a watershed segmentation. Particle analysis is conducted on the mask to identify and select nuclei based on predefined size and circularity parameters. These regions of interest (ROIs) are then used to measure p53 signal intensity within the p53 channel (mean fluorescence intensity; MFI), and the results are displayed.

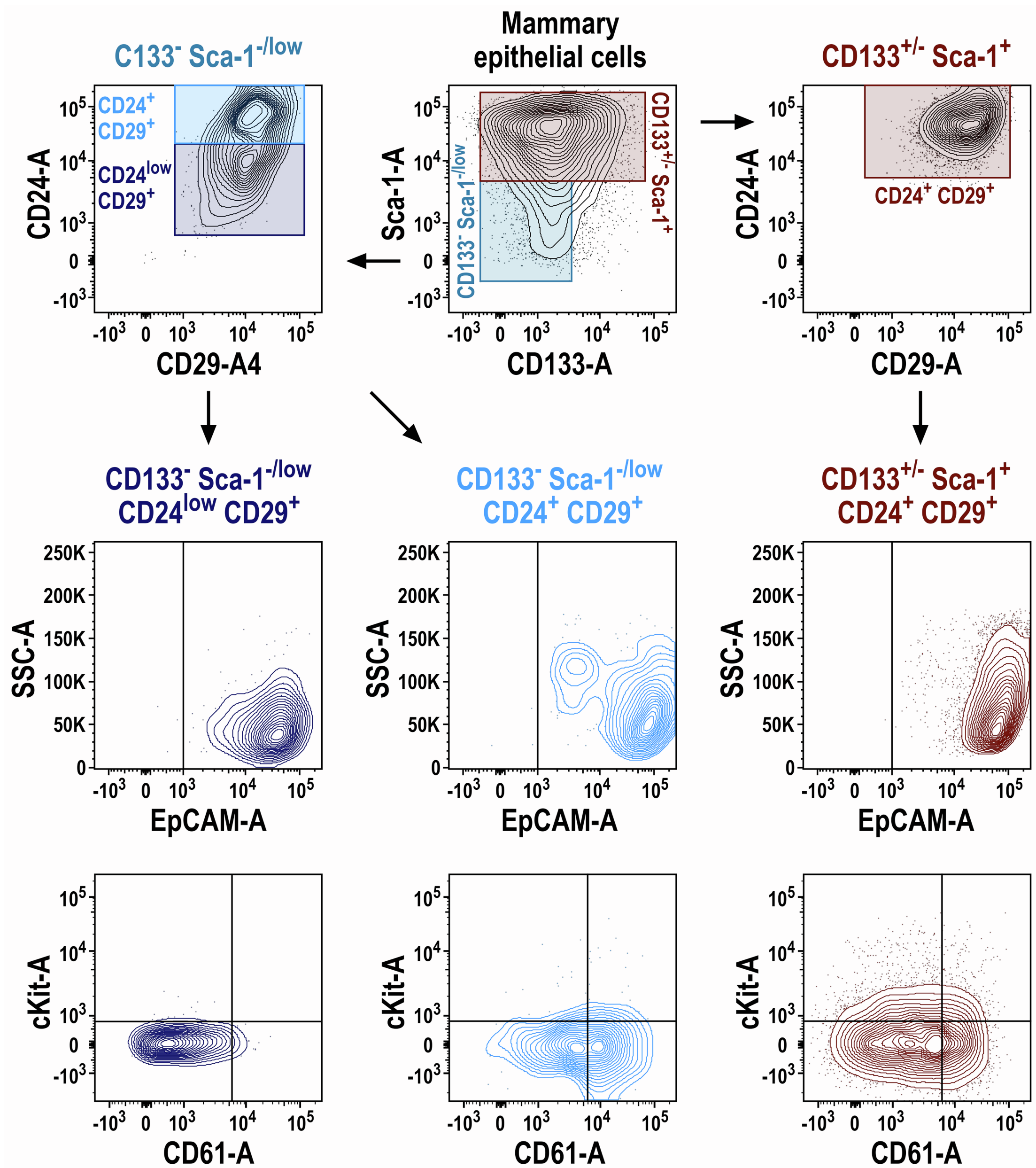

Figure S1. Cellular heterogeneity of murine mammary epithelial cells in 3D culture
Flow cytometric analysis of dissociated organoids stained with antibodies against surface proteins CD24, CD29, CD61, CD133, c-Kit, Sca-1 and EpCAM. Basal lineages and mammary stem cells (CD24^{low}CD29^{+}CD133^{-}EpCAM^{+}Sca-1$^{-/low}$) are depicted in dark blue, while luminal progenitors (CD24^{+}CD29^{+}CD133$^{+/-}$EpCAM^{high}Sca-1^{+}CD61^{+}cKit$^{+/-}$) and fully differentiated ductal and alveolar cells (CD24^{+}CD29^{+}CD133$^{+/-}$EpCAM^{high}Sca-1^{+}CD61^{-}cKit$^{-/low}$) are indicated in brown. Light blue represents a mixture of epithelial lineages.

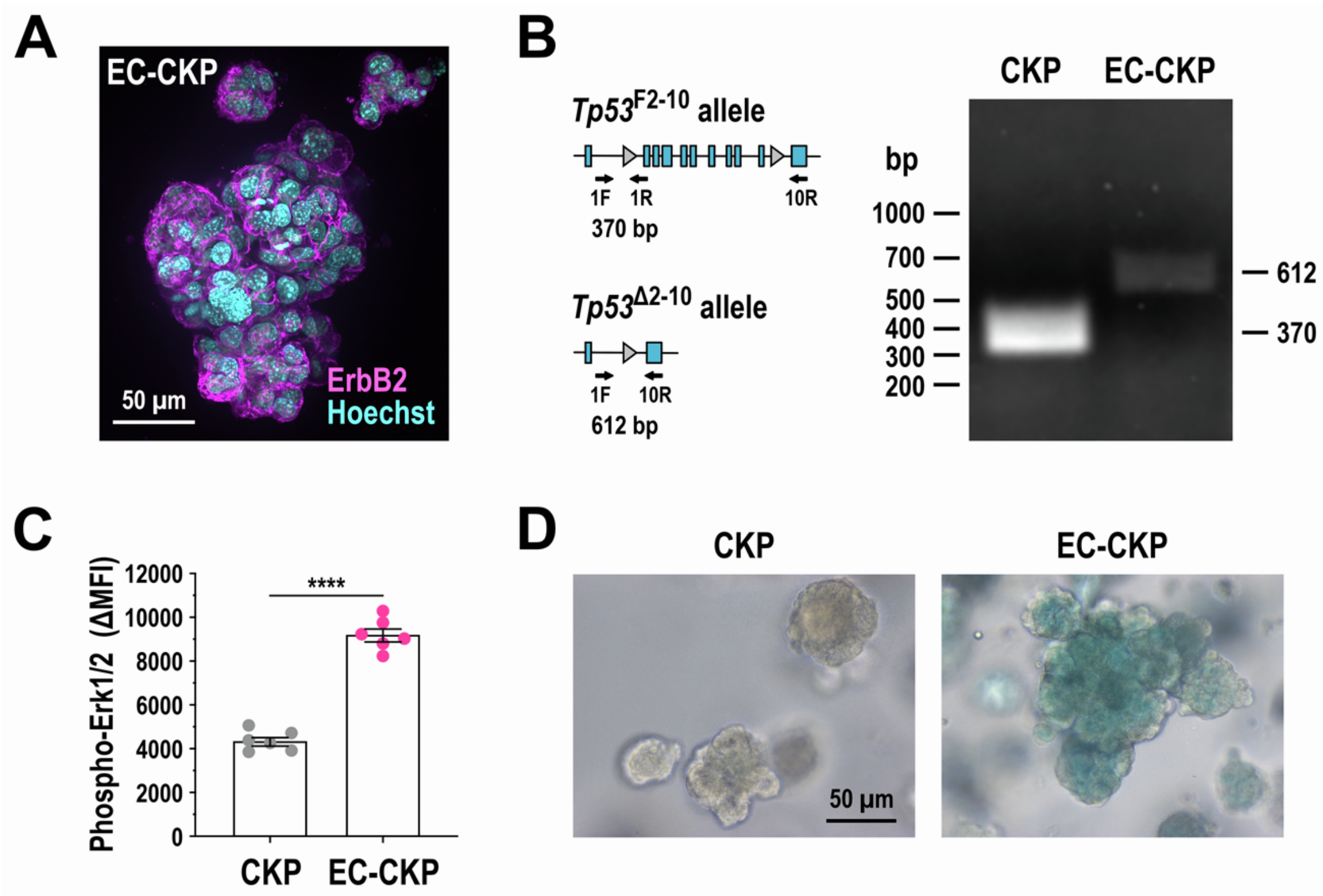

Figure S2. Genetic driver mutations in EC-CKP cells.
(A) Analysis of EC-CKP organoids by immunofluorescence microscopy. Organoids were fixed in Matrigel, stained with an ErbB2-specific antibody (magenta) and Hoechst dye (blue), and analyzed via confocal microscopy. Shown is a maximum intensity projection processed from images of a representative organoid. Scale bar: 50 µm. (B) Schematic representation of the PCR analysis detecting the *Tp53*$^{F2-10}$ allele with LoxP sequences (gray arrowheads) in introns 1 and 10, and the recombined *Tp53*$^{\Delta 2-10}$ allele lacking exons 2 to 10 (blue rectangles) after Cre-mediated recombination (left panel). PCR was performed with primers 1F, 1R, and 10R (black arrows), generating specific products of 370 base pairs (bp) in the case of unmodified CKP organoids, and 612 base pairs in the case of ErbB2- and Cre-expressing EC-CKP organoids carrying the recombined *Tp53*$^{\Delta 2-10}$ allele (right panel). (C) Flow cytometric analysis of phosphorylated Erk1/2. Organoids were dissociated, fixed, permeabilized, and then subjected to staining with a phospho-Erk1/2-specific antibody followed by species-specific fluorochrome-conjugated secondary antibody. Mean values ± SEM of delta mean fluorescence intensities (ΔMFI) from six independent experiments are shown. ****, $p < 0.0001$. (D) Enzymatic activity of the β-Geo reporter linked to Kras^{G12V} expression detected by X-Gal staining of CKP and EC-CKP organoids in Matrigel. Scale bar: 50 µm.

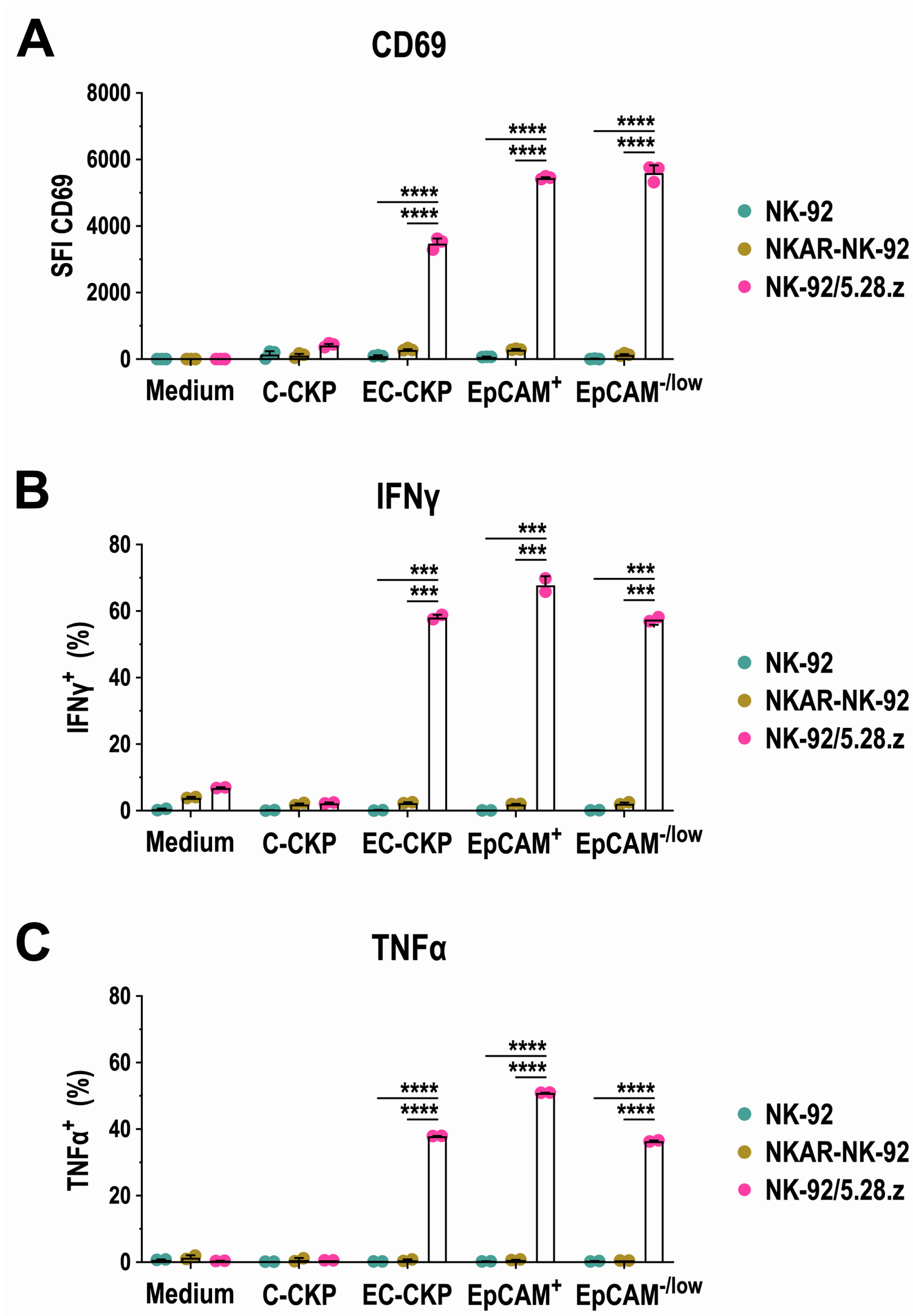

Figure S3. Activation of ErbB2-specific NK-92/5.28.z cells by *in vivo* passaged organoids
(A) Expression of the activation marker CD69 by NK-92/5.28.z cells upon co-culture with dissociated C-CKP or EC-CKP organoids from continuous *in vitro* culture, or EpCAM^{+} and EpCAM$^{-/low}$ EC-CKP tumor cells from the second *in vivo* passage was analyzed after 4 hours by flow cytometry using a CD69-specific antibody (SFI, specific fluorescence intensity). Parental NK-92 cells and NKAR-NK-92 cells expressing a CAR specific for human NKG2D ligands were included for comparison. NK cells incubated in the absence of target cells (medium) served as control. (B, C) Cytokine expression by NK-92/5.28.z cells after co-incubation with target cells for 4 hours as indicated in (A) was analyzed by intracellular staining. Percentages of IFNγ- (B) and TNFα-positive cells (C) were determined by flow cytometry. Mean values ± SD of n=3 (A) or n=2 (B, C) biological replicates are shown. ****, $p < 0.0001$; ***, $p < 0.001$.

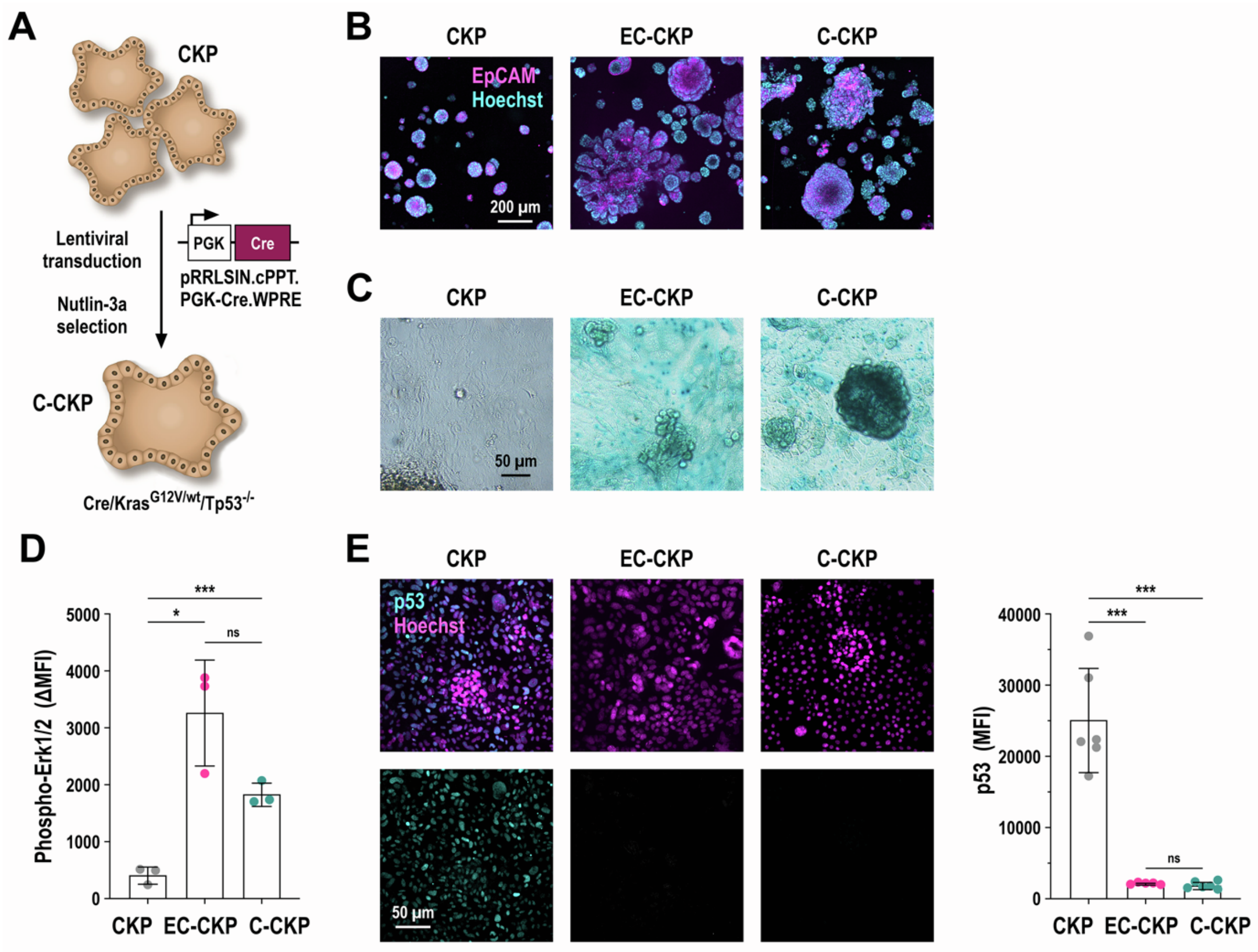

Figure S4. Establishment of mammary organoids with neoplastic driver mutations that lack ErbB2 expression

(A) For conditional activation of oncogenic KRas^{G12V} and knockout of p53 without concomitant ErbB2 expression, mammary organoids from the CKP mouse were modified via lentiviral transduction to express Cre recombinase, and selected for p53 knockout cells in medium containing 10 µM Nutlin-3a (C-CKP). (B) Morphology of newly formed CKP, EC-CKP and C-CKP organoids in Matrigel was assessed by immunofluorescence microscopy five days after seeding of fully dissociated organoid cells. Cells were stained with anti-EpCAM antibody (magenta) and counterstained with Hoechst dye (cyan). Z-sections were obtained by confocal microscopy and are presented as maximum intensity projections. Scale bar: 200 µm. (C) X-Gal staining of 2D cultures derived from parental CKP and genetically modified EC-CKP and C-CKP organoids. Scale bar: 50 µm. (D) Flow cytometric analysis of phosphorylated Erk1/2 proteins in CKP, EC-CKP and C-CKP organoids. Single-cell suspensions were fixed, permeabilized, and stained with phospho-ERK1/2-specific antibody, followed by fluorochrome-labeled secondary antibody. Mean values ± SD of delta mean fluorescence intensities (ΔMFI) from three independent experiments are shown. ***, $p < 0.001$; *, $p < 0.05$; ns, $p \geq 0.05$. (E) Expression of p53 by mammary organoids was analyzed by immunofluorescence microscopy. Dissociated CKP, EC-CKP and C-CKP organoids were cultured as 2D monolayers, fixed, permeabilized, and then stained with a p53-specific antibody, followed by fluorochrome-conjugated secondary antibody (cyan) and counterstaining with Hoechst dye (magenta). Shown are representative maximum intensity projections of z-sections (left panels). Scale bar: 50 µm. Mean fluorescence intensities (MFI) were determined using ImageJ by processing Hoechst channel images to create a binary mask, allowing quantification of p53 signals in the nuclei (right panel). Mean values ± SEM determined from at least five independent microscopic images of each cell type are shown. ***, $p < 0.001$; ns, $p \geq 0.05$.

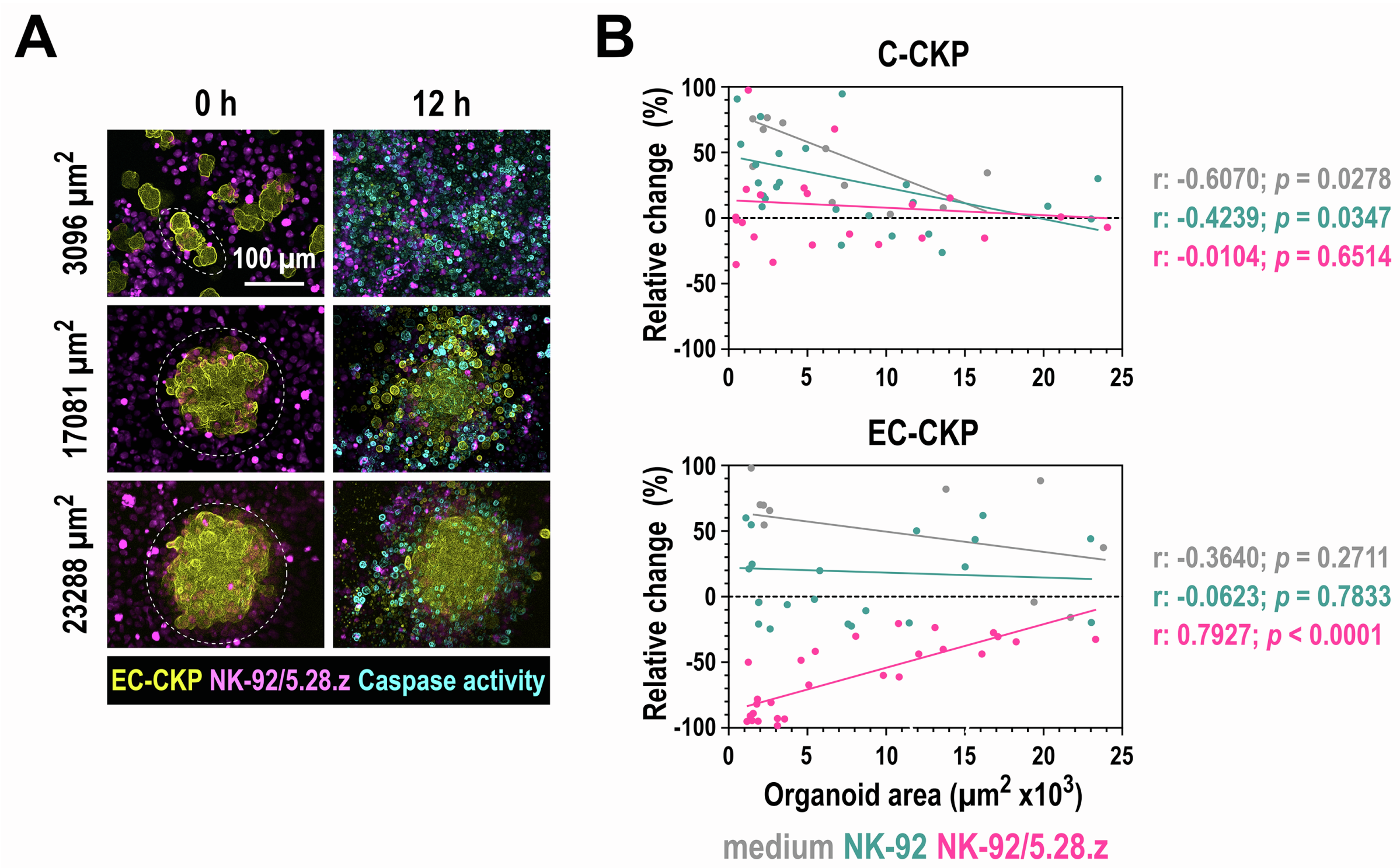

Figure S5. Size-dependent lysis of mammary organoids by CAR-NK cells
(A) For microscopic analysis of target cell killing, ErbB2-expressing EC-CKP organoids carrying a Luc2-sfGFP marker gene construct (yellow) were co-cultured with NK-92/5.28.z cells (magenta) for 12 hours in the presence of a fluorescent caspase-3/7 substrate (cyan). Shown are processed maximum intensity projections (MIP) of representative organoids of small, medium and large size (3096, 17081 and 23288 μm^2) imaged at 0 and 12 hours. Scale bar: 100 µm. (B) For quantitative analysis of size-dependent lysis of ErbB2-negative C-CKP and ErbB2-expressing EC-CKP organoids by NK-92/5.28.z and parental NK-92 cells, MIP-based hyperstacks were generated from microscopic images, and MFI values of the sfGFP signal at the beginning (0 h) and end (12 h) of the experiment were determined using ImageJ software. The relative change in signal intensity after 12 hours was calculated for each organoid and plotted against organoid size (μm^2). Organoids kept without NK cells served as control (medium). Shown are scatter plots of individual organoids from five independent experiments with corresponding linear regressions and correlation coefficients (r).

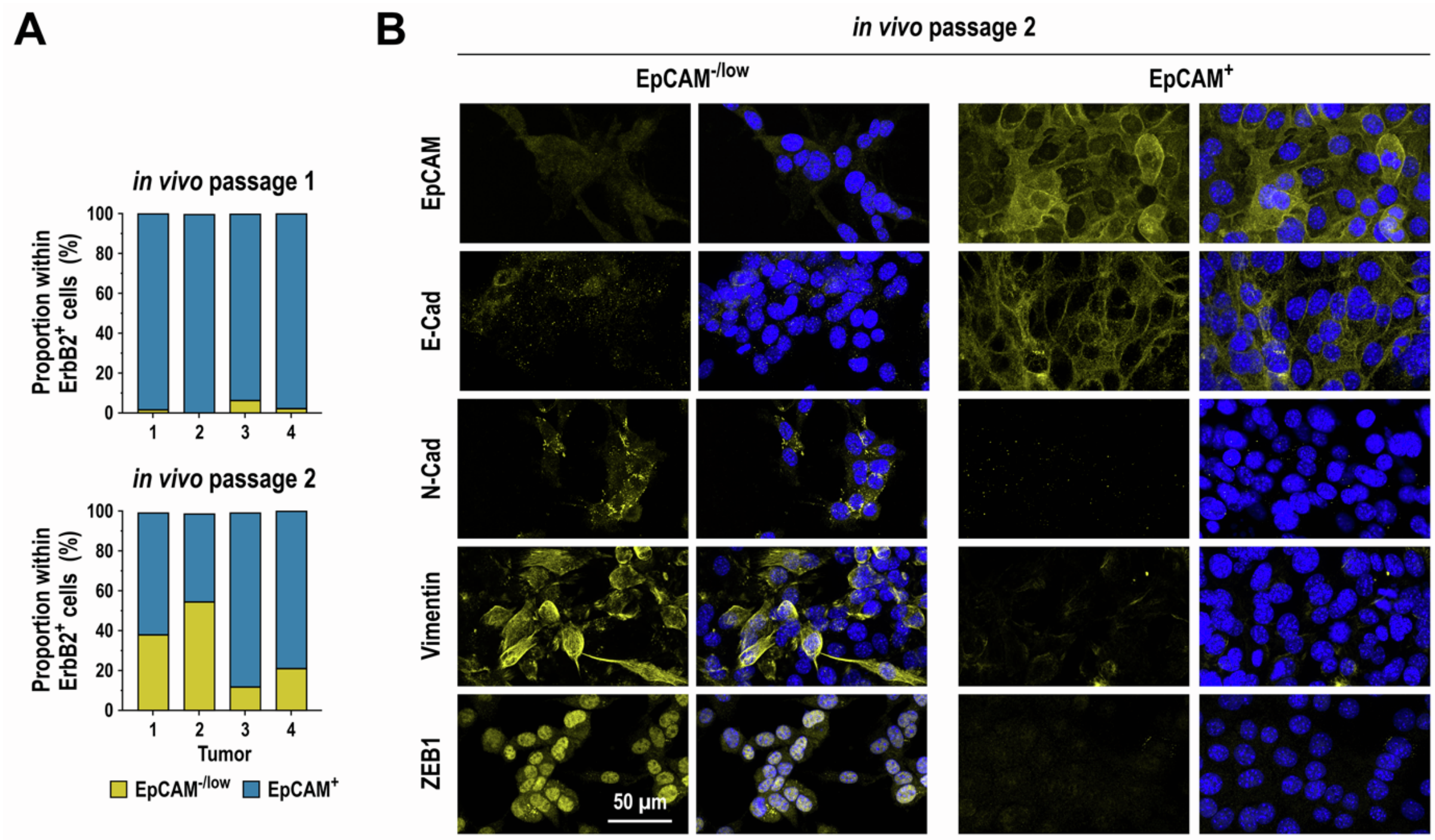

Figure S6. EMT phenotype of *in vivo* passaged organoids
(A) Single-cell suspensions of EC-CKP tumor organoid cultures after *in vivo* passage 1 (top) or 2 (bottom) were analyzed for EpCAM surface expression using flow cytometry. Data are shown as percentage of $EpCAM^{-/low}$ and $EpCAM^{+}$ cells within the ErbB2-positive population for organoids derived from four individual tumors. (B) Sorted $EpCAM^{-/low}$ and $EpCAM^{+}$ cells from *in vivo* passage 2 growing as 2D cultures were fixed, permeabilized and stained with antibodies specific for EpCAM, E-Cadherin (E-Cad), N-Cadherin (N-Cad), Vimentin and ZEB1, followed by species-specific fluorochrome-conjugated secondary antibodies. Counterstaining was performed using Hoechst dye. Shown are representative maximum intensity projections depicting the EMT markers (yellow; left panels) and merged images including the Hoechst staining (blue; right panels). Scale bar: 50 µm.

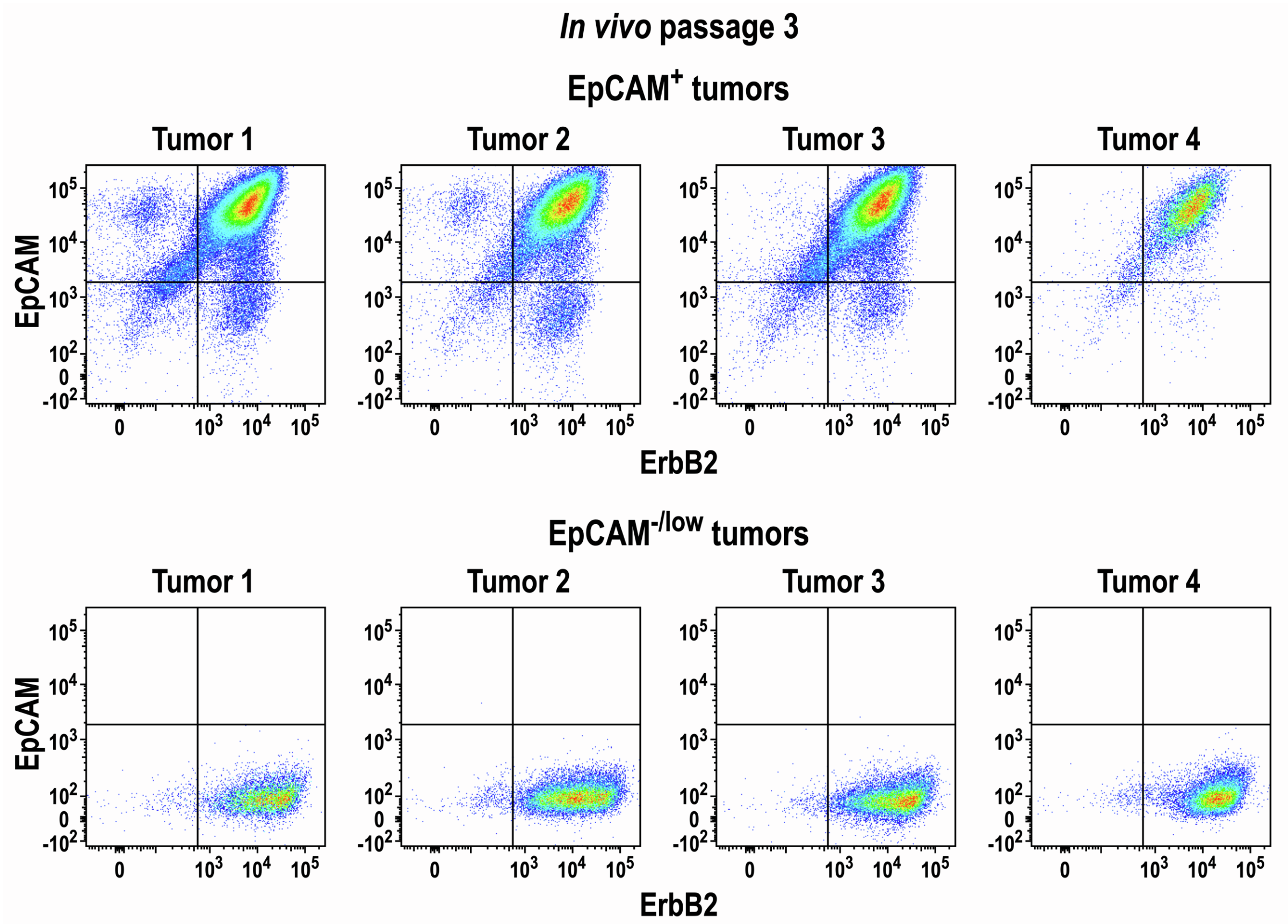

Figure S7. ErbB2 expression in tumors from *in vivo* passage 3
Subcutaneous tumors from the experiment shown in Figure 5E were excised, dissociated into single-cell suspensions and expanded for two weeks before flow cytometric analysis with EpCAM-specific and ErbB2-specific antibodies. Data for tumors from four individual animals of each group are shown.

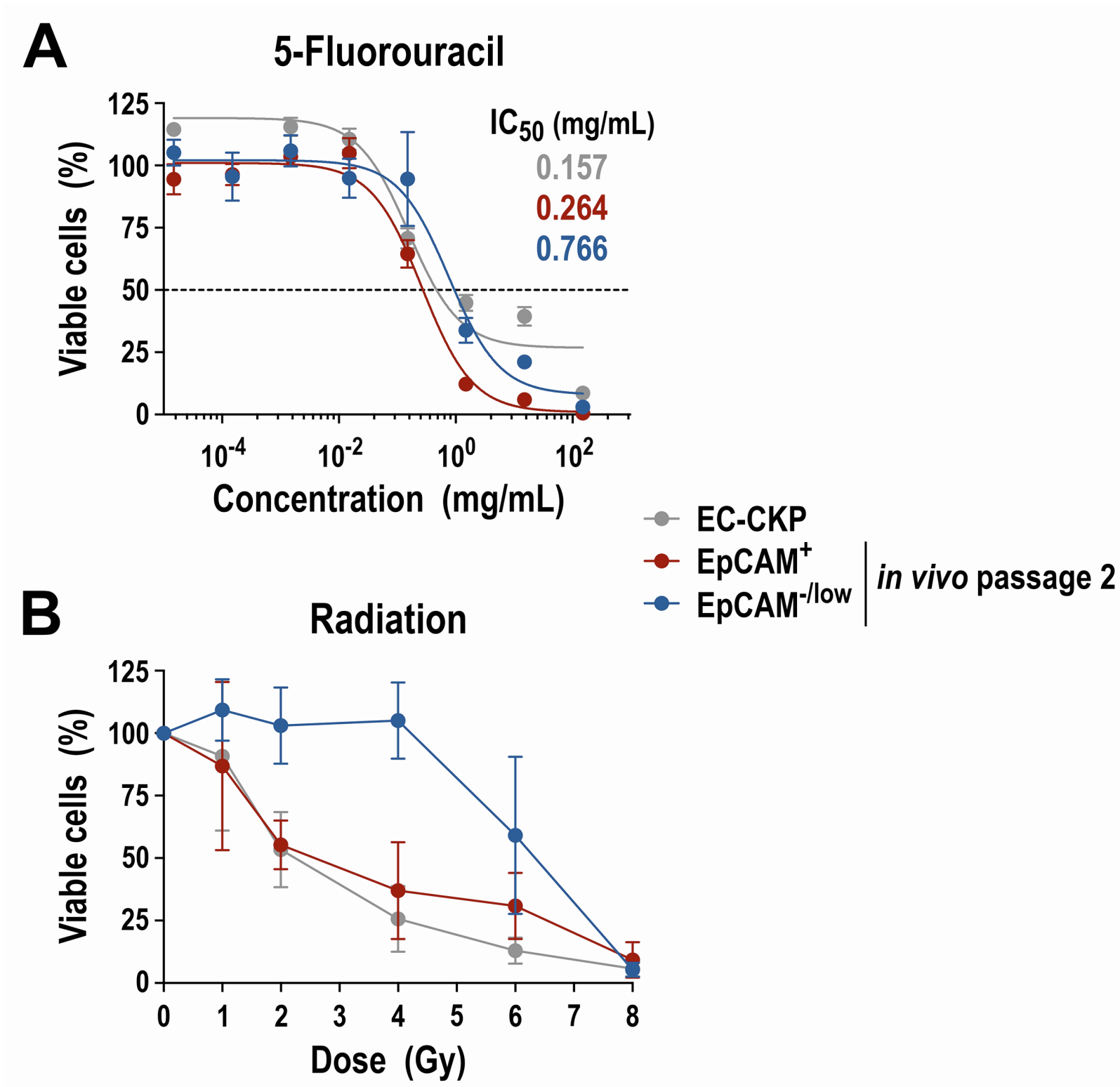

Figure S8. Sensitivity of *in vivo* passaged organoids to chemotherapy and radiation

(A) Single-cell suspensions of *in vitro* cultured EC-CKP organoids (gray) and *in vivo* passaged EpCAM^{+} (red) and EpCAM$^{-/low}$ cells (blue) tumor organoids were seeded in 96-well plates, and increasing concentrations of 5-fluorouracil were added the following day. After 5 days, cell viability was evaluated using a resazurin-based assay. For this, medium was replaced with resazurin solution (44 µM in fresh culture medium), and fluorescence was measured after 1-2 hours of incubation with a SpectraMax iD3 microplate reader (excitation: 530 nm, emission: 590 nm, integration time: 100 ms). The proportion of viable cells was calculated based on fluorescence values obtained from untreated and staurosporine-treated controls (5 µM). Mean values ± SD of n=3 technical replicates are shown. IC_{50} values were determined from dose response curves calculated using GraphPad Prism. (B) Single-cell suspensions of organoids as described in (A) were irradiated at doses of 2, 4, 6 and 8 Gy using a BioBeam 2000 GM irradiation system, followed by seeding in 12-well plates. After 7 days of culture, the proportion of viable cells compared to untreated controls was determined using a crystal violet assay. Cells were fixed with 4% paraformaldehyde and stained with of 0.5% crystal violet in 20% methanol. Cell-bound crystal violet was solubilized for 30 minutes using Sorenson's buffer (0.1 M sodium citrate, 50% EtOH, pH 4.2), and absorbance at 540 nm was measured using a SpectraMax iD3 microplate reader. Mean values ± SD of n=4 technical replicates are shown.

Supplemental Movies

Movies S1 and S2. Cytotoxic activity of CAR-NK cells against neoplastic mammary organoids
ErbB2-negative C-CKP (Movie S1) and ErbB2-expressing EC-CKP organoids (Movie S2) carrying a Luc2-sfGFP marker gene construct (yellow) were co-cultured with NK-92/5.28.z cells (magenta) for 12 hours in the presence of a fluorescent caspase-3/7 substrate (cyan). Microscopic images were acquired every 15 minutes, maximum intensity projections were derived, and assembled into MPEG-4 movies. The time stamp indicates minutes:seconds. Individual images taken at 0, 180, 360, 540 and 705 minutes of the experiment are shown in Figure 4C.
