# Supplementary material for: ErbB2/HER2-targeted CAR-NK cells eliminate breast cancer cells in an organoid model that recapitulates tumor progression
Source: Mol Ther. 2025 Apr 24;33(8):3559–75. doi: 10.1016/j.ymthe.2025.04.033 (PMC12461661; doi:10.1016/j.ymthe.2025.04.033)
Supplement: Supplementary file 2 [file mmc4.pdf]

Molecular Therapy
Original Article

ErbB2/HER2-targeted CAR-NK cells eliminate breast cancer cells in an organoid model that recapitulates tumor progression

Jasmin Röder,[1,2] Tijna Alekseeva,[1] Anne Kiefer,[1] Ines Kühnel,[1] Maren Prüfer,[1] Congcong Zhang,[1] Malena Bodden,[1] Sebastian Rosigkeit,[3] Anja Waldmann,[1] Torsten Tonn,[2,4] Ernesto Bockamp,[3] Stefan Stein,[1] and Winfried S. Wels[1,2,5]

[1]Georg-Speyer-Haus, Institute for Tumor Biology and Experimental Therapy, 60596 Frankfurt, Germany; [2]Frankfurt Cancer Institute, Goethe University, 60596 Frankfurt, Germany; [3]Institute of Translational Immunology and Research Centre for Immunotherapy, Medical Center of the Johannes Gutenberg University, 55131 Mainz, Germany; [4]Institute for Transfusion Medicine and Immunohematology, Goethe University, Frankfurt and Red Cross Blood Donation Service Baden-Württemberg-Hessen, 60528 Frankfurt, Germany; [5]German Cancer Consortium (DKTK), partner site Frankfurt/Mainz, 60596 Frankfurt, Germany

Chimeric antigen receptor-engineered NK cells hold promise for adoptive cancer immunotherapy. In one such approach, the ErbB2 (HER2)-specific CAR-NK cell line NK-92/5.28.z is under investigation as an off-the-shelf therapy in a phase I trial in glioblastoma patients. To evaluate activity of NK-92/5.28.z cells against ErbB2-positive breast cancer, here we developed an organoid model derived from CKP mice that allows conditional activation of oncogenic driver mutations. Expression of ErbB2 and Cre recombinase in CKP mammary epithelial cells induced malignant transformation, with the resulting EC-CKP cells characterized by neoplastic morphology, loss of p53, and constitutive activation of the MAP kinase pathway. NK-92/5.28.z cells demonstrated potent CAR-mediated cytotoxicity against EC-CKP organoids, with tumor cell lysis dependent on exposure time and organoid size. *In vivo* passaging of EC-CKP organoids revealed cellular plasticity and induced an EMT phenotype associated with increased resistance to standard therapies. Importantly, NK-92/5.28.z cells retained high and specific cytotoxicity against these breast cancer cells *in vitro* and in an aggressive organoid-based *in vivo* mouse model that reflects advanced-stage disease. Our data highlight the therapeutic potential of NK-92/5.28.z cells against ErbB2-positive breast cancer, supporting their further development toward clinical application.

INTRODUCTION

Breast cancer is the most common malignancy affecting women worldwide and a leading cause of cancer-related mortality,[1] with progression, invasiveness, and therapy response of this heterogeneous disease depending on its histological and molecular characteristics. Approximately 20%–25% of patients with invasive breast cancer exhibit elevated levels of ErbB2 (HER2), an EGFR-related receptor tyrosine kinase that regulates cell growth and has transforming potential when overexpressed or mutated.[2] Overexpression of ErbB2 correlates with higher breast cancer aggressiveness, and has historically been associated with an unfavorable clinical outcome. This has changed with the introduction of targeted therapies such as the monoclonal antibodies trastuzumab and pertuzumab, tyrosine kinase inhibitors and antibody-drug conjugates, which are part of current treatment algorithms and have significantly improved progression-free and overall survival for patients with ErbB2-positive disease.[3–5] Nevertheless, initial treatment failure and acquired resistance to these reagents justify ongoing efforts to develop alternative ErbB2-targeted therapeutics, which also include different types of immunotherapies.[3,6]

For adoptive cancer immunotherapy, natural killer (NK) cells have emerged as a promising cell type due to their innate ability to recognize and eliminate malignant cells, and the possibility to apply them as an allogeneic off-the-shelf therapeutic without the risk of inducing severe graft-versus-host disease.[7–9] As in the case of T lymphocytes, the antitumor activity of NK cells can be significantly enhanced by engineering them with chimeric antigen receptors (CARs), thereby complementing the NK cells' natural cytotoxicity with targeted, antigen-specific tumor cell killing.[10–12] Promising results have been reported from a recent phase I/II trial with cord blood-derived NK cells carrying a CD19-specific CAR as a therapy for B cell malignancies, with response rates comparable with those obtained after treatment with CAR-T cells, but without adverse effects such as cytokine release syndrome or neurotoxicity typical for the latter.[13,14]

We previously generated the ErbB2-specific CAR-NK cell line NK-92/5.28.z, which demonstrated high and selective activity against different types of solid tumors in experimental models.[15–17] This has enabled clinical translation, with NK-92/5.28.z cells currently being evaluated as an allogeneic off-the-shelf CAR-NK cell therapeutic

Received 12 December 2024; accepted 22 April 2025;
https://doi.org/10.1016/j.ymthe.2025.04.033.

Correspondence: Winfried S. Wels, Georg-Speyer-Haus, Institute for Tumor Biology and Experimental Therapy, Paul-Ehrlich-Straße 42-44, 60596 Frankfurt am Main, Germany.
E-mail: wels@gsh.uni-frankfurt.de

Published by Elsevier Inc. on behalf of The American Society of Gene and Cell Therapy.
This is an open access article under the CC BY license (http://creativecommons.org/licenses/by/4.0/).

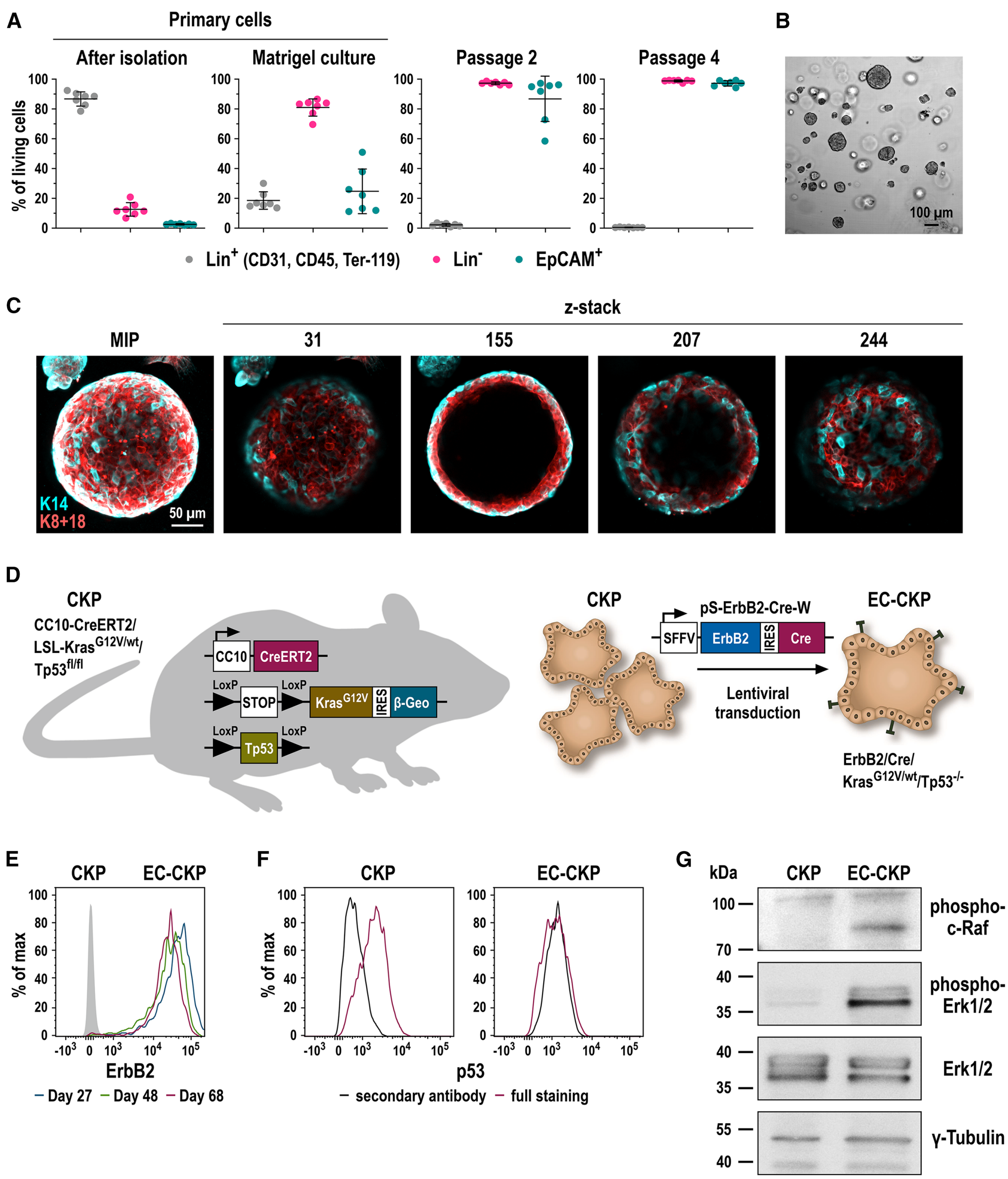

Figure 1. Establishment of murine mammary organoids as a model for ErbB2-positive breast cancer

(A) Composition of primary cells after isolation from mammary fat pads and subsequent passages as 3D organoid cultures. Percentages of cells expressing lineage markers (CD31, CD45, Ter-119), and EpCAM were determined by flow cytometric analysis of cultures established from seven independent donor mice. (B) Representative microscopic image of organoid morphology after achieving a homogeneous epithelial cell culture (> passage 4). Scale bar, 100 μm. (C) Confocal microscopy analysis of cytokeratin K8/18 (red) and K14 (cyan) expression by cells of a representative organoid cultured in Matrigel. Immunofluorescence images of a maximum intensity projection (MIP; left) and four optical sections are shown, with K14 predominantly expressed by the outer cell layer and K8/18 by cells facing the lumen. Scale bar, 50 μm. (D) Generation of ErbB2-expressing mammary organoids carrying genetic driver mutations. Organoids were derived from the CKP mouse model (left) that allows conditional activation of oncogenic *Kras*^{G12V} and knockout of *Tp53*, and further modified via lentiviral transduction to express ErbB2 and Cre recombinase (EC-CKP) as schematically shown on the right. (E) ErbB2-Cre-transduced organoid cells were enriched by flow cytometric cell sorting, and stability of ErbB2 surface expression over time was confirmed by flow

(legend continued on next page)

in a phase I trial in patients with recurrent ErbB2-positive glioblastoma (CAR2BRAIN; NCT03383978).[18,19] Similar to brain tumors, NK-92/5.28.z cells may also become applicable for the treatment of more prevalent ErbB2-positive malignancies, with activity of the CAR-NK cells against breast cancer so far demonstrated in *in vitro* tissue culture models with established tumor cell lines.[15,20,21] Nevertheless, to enhance the significance of preclinical studies and aid later clinical translation of CAR-NK cell immunotherapies, more complex experimental models are required that better reflect breast cancer pathophysiology and the physical barriers of a tumor tissue. In this respect, the development of organoid technology constitutes a major advancement.[22] Organoids are characterized by organotypic differentiation and compartmentalization, ensuring that established 3D cultures phenotypically and functionally correspond well with the original tissue, thereby enabling a physiologically relevant analysis of therapeutic interventions.[23,24]

Starting out from healthy murine mammary epithelial cells, here we developed an organoid model of ErbB2-positive breast carcinoma characterized by a loss of p53 and signaling pathway alterations typical for this cancer subtype. Utilizing organoid cultures which replicate different stages of tumor development and increasing aggressiveness, we analyzed the therapeutic activity of ErbB2-specific NK-92/5.28.z cells against these tissue-like 3D structures *in vitro*, and then investigated the efficacy of the CAR-NK cell immunotherapy in an organoid-based *in vivo* model that reflects advanced-stage disease.

RESULTS

Generation of a murine organoid model of ErbB2-positive breast cancer

To investigate the therapeutic potential of CAR-NK cells in a 3D *in vitro* model of ErbB2-positive breast cancer, first a robust protocol for the generation of mammary organoids from primary tissue cells of CC10-CreERT2 $Kras^{LSLG12Vgeo/WT}$ $Tp53^{fl/fl}$ (CKP) mice was established. This genetically engineered mouse model allows conditional activation of oncogenic $Kras^{G12V}$ and simultaneous inactivation of *Tp53*.[25] Cell mixtures were derived from enzymatically digested abdominal mammary glands of female animals, embedded in Matrigel, passaged weekly or biweekly, and analyzed by flow cytometry (Figure 1A). Initially, cultures contained an average of 2.59% ± 0.46% EpCAM-positive cells, followed by a marked enrichment of epithelial cells over successive passages and the establishment of pure epithelial cell organoid cultures at passage 4 (Figures 1A and 1B). The resulting 3D structures exhibited organotypic compartmentalization, characterized by an inner layer of K8/K18-positive luminal cells, surrounded by a basal myoepithelium marked by K14 expression (Figure 1C). Flow cytometric analysis confirmed the presence of different epithelial cell lineages, identifying luminal cells ($CD24^+CD29^+CD133^{+/-}$ $EpCAM^{high}Sca\text{-}1^+$) and basal cells ($CD24^{low}CD29^+CD133^-EpCAM^+$ $Sca\text{-}1^{-/low}$) (Figure S1), with the latter pattern also typical for mammary stem cells.[26] Additionally, differences in the expression of CD61 and c-Kit indicated the presence of both, luminal progenitors ($CD24^+CD29^+CD133^{+/-}EpCAM^{high}Sca\text{-}1^+CD61^+c\text{-}Kit^{+/-}$) and fully differentiated ductal and alveolar cells ($CD24^+CD29^+CD133^{+/-}$ $EpCAM^{high}Sca\text{-}1^+CD61^-c\text{-}Kit^{-/low}$), corroborating the organotypic cellular composition of the model.

For expression of ErbB2, activation of $Kras^{G12V}$ and inactivation of *Tp53* in the mammary organoids, dissociated CKP organoids were transduced with an MLV-pseudotyped lentiviral vector encoding human ErbB2 and Cre recombinase (Figure 1D). Modified ErbB2/Cre cells (EC-CKP) were then enriched by flow cytometric cell sorting, and exhibited stable ErbB2 surface expression during subsequent organoid culture for more than 2 months (Figures 1E and S2A). Genetic inactivation of *Tp53* in EC-CKP cells was verified by PCR analysis of genomic DNA (Figure S2B), and loss of p53 protein expression was confirmed by flow cytometry (Figure 1F). Elevated levels of phosphorylated Erk1/2 and c-Raf were found in EC-CKP but not parental CKP organoids (Figures 1G and S2C), which is indicative of constitutive MAPK pathway activity due to $Kras^{G12V}$ expression. Additionally, transgene expression in EC-CKP organoids was confirmed by detecting enzymatic activity of the β-Geo reporter linked to the $Kras^{G12V}$ allele (Figure S2D).

These data demonstrate successful establishment of mammary organoids displaying a loss of p53 and signaling pathway alterations that are characteristic for ErbB2-positive breast cancer.

Neoplastic features of EC-CKP organoids

Next, we examined morphological and molecular changes of EC-CKP organoids indicative of malignant transformation. Parental CKP and ErbB2/Cre-modified EC-CKP organoids were fully dissociated, seeded in Matrigel at equal cell densities, and analyzed microscopically after 5 days to assess morphological differences. While newly formed CKP organoids displayed a cystic phenotype, EC-CKP organoids developed highly branched structures with a significantly increased surface area (Figure 2A). Nuclear morphology was investigated in 2D cell cultures derived from the organoids. Thereby, delineating cell boundaries with an anti-EpCAM antibody and staining of nuclei with Hoechst dye revealed features of EC-CKP cells commonly associated with tumor cells, including markedly expanded cell bodies, multinucleated cells, and significantly enlarged nuclei when compared with parental CKP cells (Figure 2B). To analyze proliferative activity, sections of intact organoids were examined by immunohistochemical staining for expression of the proliferation marker Ki67 (Figure 2C). Imaging analysis was performed to

cytometry at days 27, 48, and 68 of continuous culture as indicated. (F) Flow cytometric analysis of p53 expression in parental CKP and genetically modified EC-CKP organoids. Single-cell suspensions were fixed, permeabilized, and stained with a p53-specific antibody, followed by fluorochrome-labeled secondary antibody (magenta). Control cells were only stained with secondary antibody (gray). (G) Whole-cell lysates from CKP and EC-CKP organoids were analyzed by SDS-PAGE and immunoblotting with antibodies specific for phospho-c-Raf, phospho-Erk1/2, and Erk1/2 as indicated. γ-Tubulin served as a loading control.

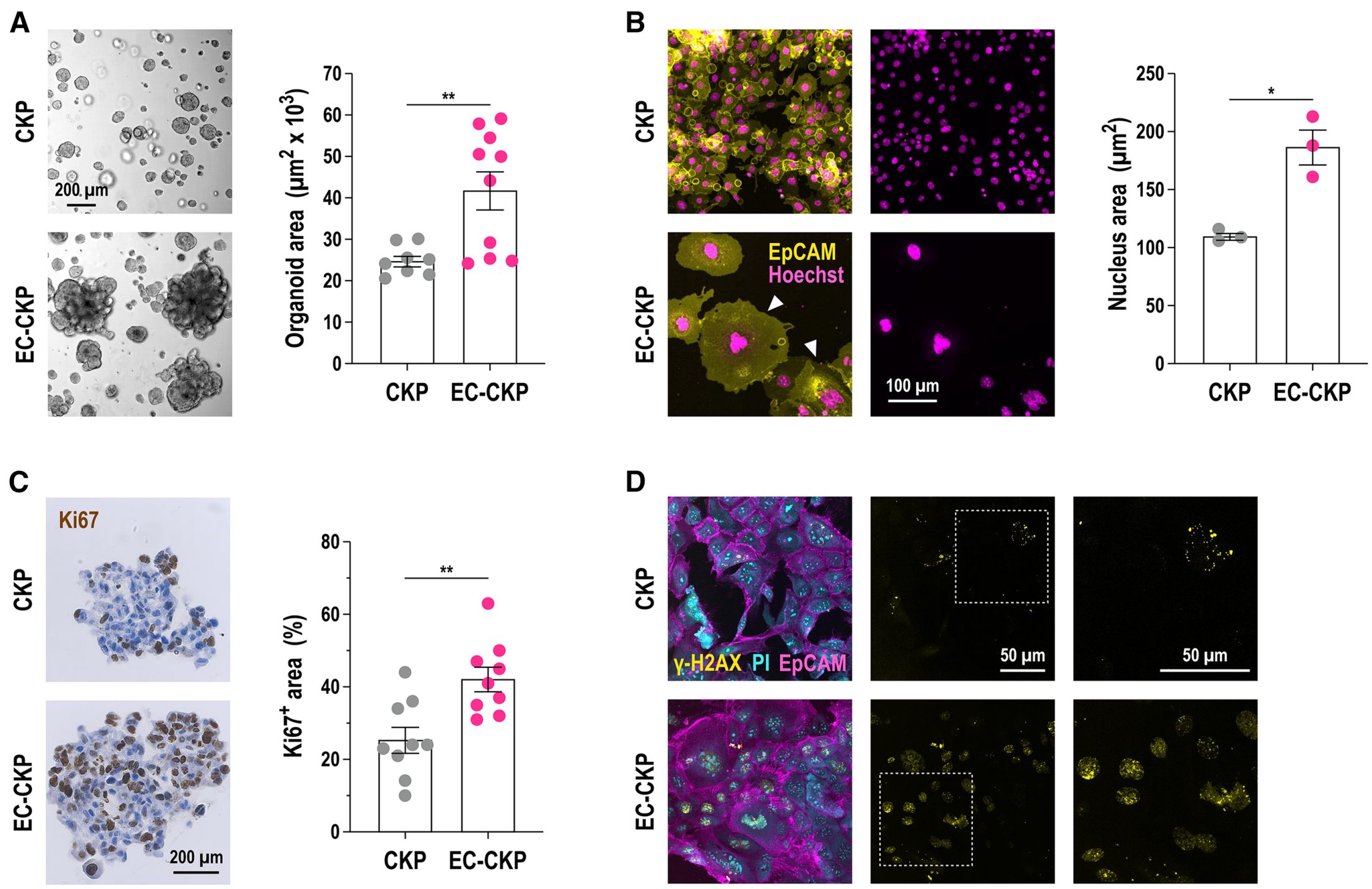

Figure 2. Morphological and molecular changes in EC-CKP organoids

(A) Determination of organoid size by microscopic analysis. CKP and EC-CKP organoids were fully dissociated, cells were re-seeded in Matrigel, and newly formed organoids were analyzed by bright-field microscopy 5 days later. Representative images are shown on the left. Scale bar, 200 μm. Organoid area was determined by processing microscopy images using ImageJ to create a binary mask enabling size quantification. Data are presented as mean ± SEM of the average organoid area from at least eight different images (right). $^{**}p < 0.01$. (B) Determination of nucleus size by immunofluorescence microscopy. Dissociated CKP and EC-CKP organoid cells were cultured as 2D monolayers for 3 days, stained with an EpCAM-specific antibody to label cell bodies (yellow), and counterstained with Hoechst dye to identify nuclei (magenta). Confocal imaging was used to obtain z-sections, presented as maximum intensity projections. Representative images are shown on the left. Multi-nucleated cells are indicated by white arrowheads. Scale bar, 100 μm. Nucleus area was determined using ImageJ by processing Hoechst channel images to create a binary mask allowing particle analysis. Data are presented as mean ± SEM of the average nucleus area from three different images. $^{*}p < 0.05$. (C) Analysis of Ki67 expression by immunohistochemistry. Representative microscopic images of CKP and EC-CKP organoids are shown on the left. Scale bar, 200 μm. Quantification of the Ki67-positive area (right) was performed using QuPath software. Total organoid area was determined, images were processed, a threshold for the Ki67 signal was set, and the percentage of Ki67-positive area within the selected total area was calculated. Data are presented as mean ± SEM of the percental Ki67-positive area from nine different images. $^{**}p < 0.01$. (D) Analysis of γ-H2AX expression by immunofluorescence microscopy. Dissociated CKP and EC-CKP organoid cells were cultured as 2D monolayers, stained with an EpCAM-specific antibody (magenta), fixed, permeabilized, and then stained with a fluorochrome-conjugated γ-H2AX-specific antibody (yellow), followed by counterstaining with propidium iodide (PI) (cyan). Shown are representative maximum intensity projections of z-sections. Scale bars, 50 μm.

quantify the total organoid area and the Ki67-positive area within each organoid, revealing an average Ki67-positive area of 26% ± 3.6% for CKP organoids, which was significantly increased to 42% ± 3.4% for EC-CKP organoids (Figure 2C). As another indicator of malignant transformation, we next assessed chromosomal instability by analyzing γ-H2AX expression as a marker for DNA double-strand breaks in 2D cell cultures derived from the organoids.[27] Indeed, immunofluorescence staining of γ-H2AX and confocal microscopy revealed a pronounced accumulation of DNA double-strand breaks in EC-CKP cells, which was not found in parental CKP cells (Figure 2D).

Collectively, the marked morphological and molecular changes observed in EC-CKP organoids upon *Tp53* knockout and constitutive activation of the MAP kinase signaling pathway are indicative of malignant transformation, suggesting these organoids as a suitable 3D *in vitro* model of ErbB2-positive breast cancer.

Specific activity of CAR-NK cells against organoid-derived mammary epithelial cells

To assess the sensitivity of EC-CKP cells to ErbB2-specific NK-92/5.28.z CAR-NK cells, we first conducted co-cultivation experiments using dissociated organoids. NK cells and mammary epithelial cells were co-incubated at different effector-to-target (E/T) cell ratios for 4 h, followed by determining target cell lysis using a flow cytometric assay (Figure 3A). As expected, neither parental NK-92 nor CAR-engineered NK-92/5.28.z cells were able to lyse ErbB2-negative CKP cells included for comparison. In contrast, while unaffected by parental NK-92 cells, ErbB2-expressing EC-CKP cells were efficiently lysed by NK-92/5.28.z cells in a dose-dependent manner,

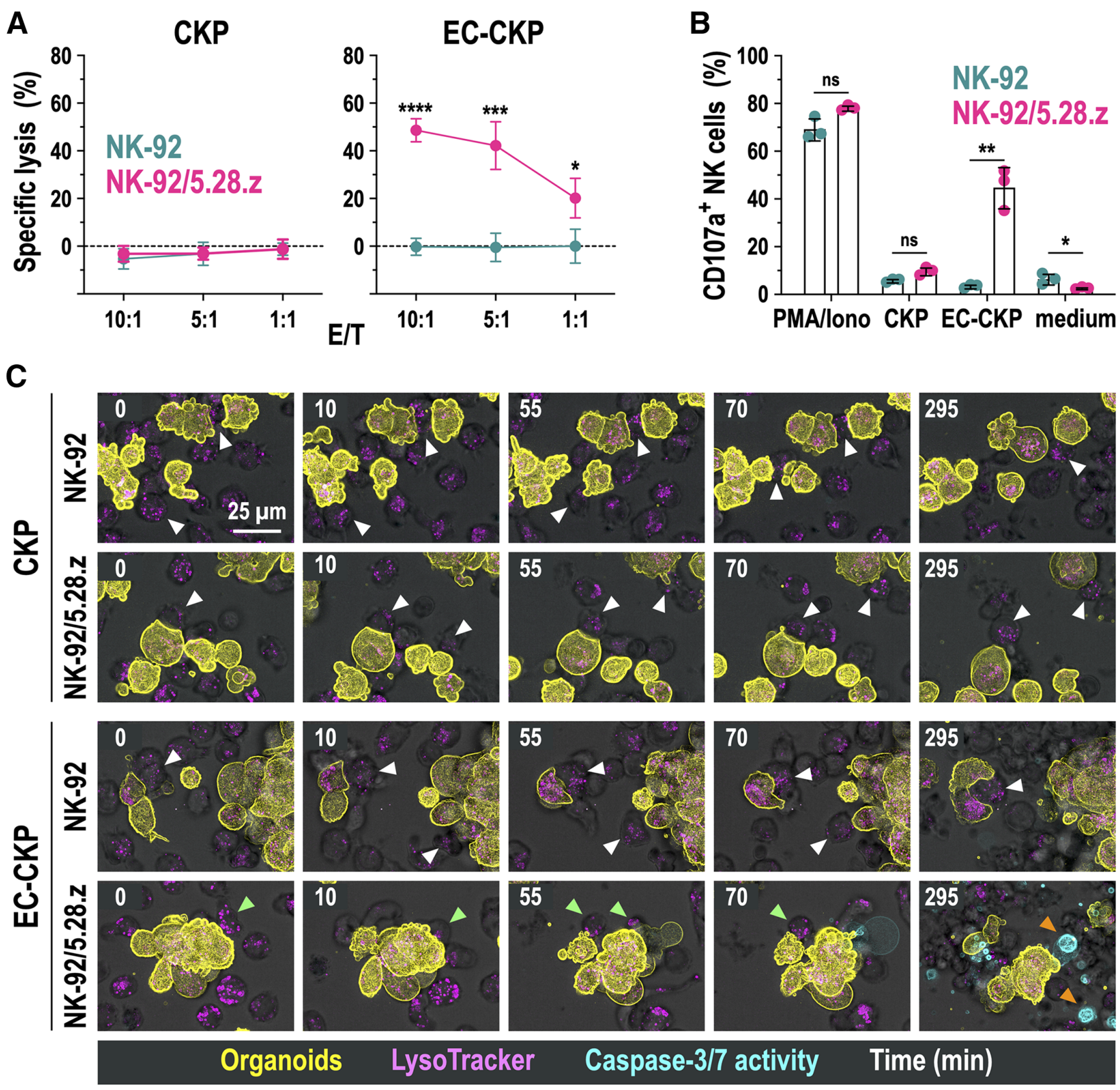

Figure 3. Cytotoxic activity of CAR-NK cells against dissociated organoids in 2D culture

(A) Dissociated CKP and EC-CKP organoids were co-incubated with CAR-engineered NK-92/5.28.z or parental NK-92 cells at the indicated effector-to-target cell (E/T) ratios for 4 h, followed by flow cytometric analysis of specific lysis. Mean values ± SEM from four independent experiments are shown. ****$p < 0.0001$, ***$p < 0.001$, *$p < 0.05$. (B) Degranulation of NK-92/5.28.z and NK-92 cells upon co-culture with dissociated CKP and EC-CKP organoid cells for 4 h was determined by flow cytometric analysis of CD107a surface expression. Control samples were kept in the absence of target cells (medium) or stimulated with PMA and ionomycin (PMA/Iono). Mean values ± SEM from three independent experiments are shown. **$p < 0.01$, *$p < 0.05$, ns, not significant, $p \geq 0.05$. (C) For microscopic analysis of cytotoxic granule exocytosis and target cell killing, dissociated CKP and EC-CKP organoid cells carrying a Luc2-sfGFP marker gene construct (yellow) were co-cultured for 5 h with LysoTracker-stained NK-92/5.28.z or NK-92 cells (magenta) in the presence of a fluorescent caspase-3/7 substrate (cyan). Microscopic images were captured every 5 min. Shown are representative maximum intensity projections of processed images from the indicated time points. Conjugates of NK and target cells without degranulation (white arrowheads), directed polarization of lytic granules (green arrowheads), and subsequent target cell lysis (orange arrowheads) are indicated. Scale bar, 25 μm.

indicating specific cytotoxicity mediated by CAR engagement. Furthermore, exposure to EC-CKP but not CKP mammary epithelial cells triggered cytotoxic granule exocytosis of the ErbB2-specific CAR-NK cells in 4 h flow cytometric degranulation experiments measuring CD107a exposure on the effector cell surface (Figure 3B). While a similar level of degranulation was induced in NK-92 and NK-92/5.28.z cells upon stimulation with phorbol 12-myristate 13-acetate (PMA) and ionomycin, granule exocytosis in parental NK-92 cells was neither provoked by contact with EC-CKP nor CKP cells, confirming CAR dependency of NK-92/5.28.z activation by the target cells. Similarly, exposure to EC-CKP but not ErbB2-negative organoid cells induced upregulation of the activation marker CD69 and production of IFN-γ and TNF-α by ErbB2-specific NK-92/5.28.z cells (Figure S3), but not by parental NK-92 or NKAR-NK-92 cells expressing a CAR specific for human NKG2D ligands not present on the murine target cells.[28]

Effector cell activity was also analyzed in microscopic time-lapse studies with dissociated Luc2-sfGFP-expressing mammary epithelial cells derived from EC-CKP and CKP organoids. Thereby, NK-92 and NK-92/5.28.z cells stained with LysoTracker to visualize lytic granules formed conjugates with both types of mammary epithelial cells. However, while granules were still randomly distributed post conjugate formation in cultures of NK-92/5.28.z with CKP, and parental NK-92 with CKP and EC-CKP cells, lytic granules in CAR-NK cells converged upon contact with ErbB2-expressing EC-CKP cells and polarized toward the immunological synapse, subsequently resulting in apoptotic target cell death indicated by fluorescent conversion of a caspase-3/7 substrate (Figure 3C).

These findings demonstrate that, unlike parental CKP cells, ErbB2-expressing and transformed EC-CKP mammary epithelial cells are susceptible to specific CAR-mediated lysis by NK-92/5.28.z cells in 2D culture systems.

Cytotoxicity of CAR-NK cells against neoplastic mammary organoids in 3D culture

To investigate potential differences in the activity of CAR-NK cells against ErbB2-expressing mammary epithelial cells growing in 2D or 3D culture, we performed luciferase-based cytotoxicity assays with EC-CKP/Luc2-sfGFP cells co-cultured as intact organoids or suspensions of dissociated cells with NK-92/5.28.z or parental NK-92 cells at different E/T ratios for 4 h. While only marginal changes in the luciferase activity of dissociated EC-CKP cells or intact organoids were noted upon exposure to NK-92 cells, specific lysis reflected by a marked reduction in luciferase activity was detected in co-cultures with ErbB2-targeted CAR-NK cells already at an E/T ratio of 1:1, which increased further at higher E/T ratios

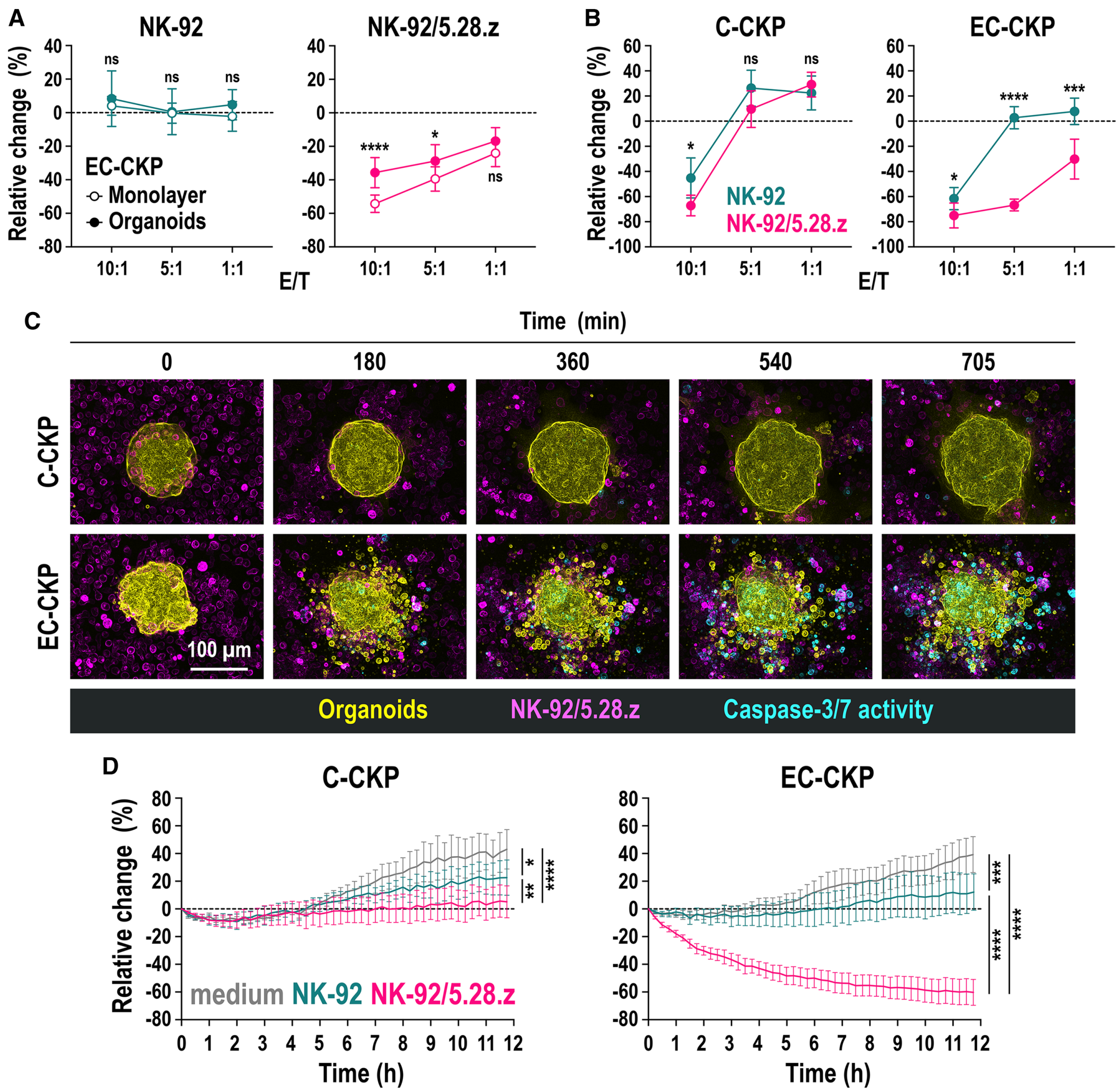

Figure 4. Cytotoxic activity of CAR-NK cells against intact mammary organoids

(A) Luc2-sfGFP-expressing EC-CKP cells growing as monolayers or organoid cultures were co-incubated with CAR-engineered NK-92/5.28.z or parental NK-92 cells at the indicated E/T ratios for 4 h. Then the relative change in bioluminescence was determined in luciferase assays. Mean values ± SD from three independent experiments are shown. ****$p < 0.0001$, *$p < 0.05$, ns, $p \geq 0.05$. (B) Intact luciferase-expressing EC-CKP organoids were co-incubated with NK-92/5.28.z or NK-92 cells at the indicated E/T ratios for 12 h. Then the relative change in bioluminescence was determined. For comparison C-CKP organoids were included, which carry the same Cre-induced driver mutations but lack ErbB2 expression. Mean values ± SD are shown ($n = 7$ independent samples analyzed for each condition). ****$p < 0.0001$, ***$p < 0.001$, *$p < 0.05$, ns, $p \geq 0.05$. (C and D) For microscopic analysis of target cell killing, intact C-CKP and EC-CKP organoids carrying a Luc2-sfGFP marker gene were co-cultured with NK-92/5.28.z or NK-92 cells for 12 h. Organoids kept without NK cells served as controls (medium). (C) Processed maximum intensity projections are shown at the indicated time points of C-CKP or EC-CKP organoids (yellow) co-cultured with NK-92/5.28.z cells (magenta) in the presence of a fluorescent caspase-3/7 substrate (cyan). Scale bar, 100 μm. (D) To determine kinetics of NK-mediated lysis of mammary organoids, for each time point MIP-based hyperstacks were generated using ImageJ software, the MFI of the sfGFP signal was calculated, and the relative change to the initial value was determined. Mean values ± SEM from five independent experiments are shown, each analyzing three to eight organoids per condition. ****$p < 0.0001$, ***$p < 0.001$, **$p < 0.01$, *$p < 0.05$.

(Figure 4A). Nevertheless, in these short-term assays cytotoxicity of NK-92/5.28.z cells against intact organoids was significantly lower than that against dissociated EC-CKP/Luc2-sfGFP cells, likely due to more limited accessibility of the target cells in complex 3D structures.

Accordingly, we next conducted long-term cytotoxicity studies with intact EC-CKP organoids. Since these cells in addition to ErbB2 carry oncogenic driver mutations not present in parental CKP cells, C-CKP organoids were instead used as a control, which are similar to EC-CKP by expressing activated $Kras^{G12V}$ and lacking p53 expression,

but are ErbB2 negative. C-CKP cells were generated by lentiviral transduction of mammary epithelial cells from CKP mice with a vector encoding Cre recombinase, followed by selection of p53-negative cells with Nutlin-3a (Figure S4A). The resulting C-CKP organoids showed the same morphological and molecular features as EC-CKP organoids, including the formation of branched structures, increased MAPK pathway activity and absence of p53 protein expression (Figures S4B–S4E). In long-term luciferase reporter assays over 12 h, Luc2-sfGFP-expressing C-CKP and EC-CKP organoids were co-cultured with NK-92/5.28.z or parental NK-92 cells (Figure 4B). Thereby, at a high E/T ratio of 10:1, ErbB2-independent lysis of both organoid lines due to natural cytotoxicity of NK and CAR-NK cells was observed. In contrast, at the lower E/T ratios of 5:1 and 1:1, ErbB2-negative C-CKP organoids were no longer lysed by NK-92 or NK-92/5.28.z cells. However, EC-CKP organoids were still readily eliminated by the ErbB2-specific CAR-NK cells, and to a much larger extent than in the prior short-term assays.

Next, we performed microscopic time-lapse experiments over 12 h to investigate dynamics and spatial processes of NK-mediated lysis of mammary organoids. Representative images captured at different time points during these experiments illustrate the interactions between fluorescently labeled NK-92/5.28.z cells and Luc2-sfGFP-expressing C-CKP and EC-CKP organoids (Figure 4C). Complete image sequences are shown as Videos S1 (C-CKP) and S2 (EC-CKP). While contact with the CAR-NK cells did not result in marked changes of ErbB2-negative C-CKP organoids (Figure 4C, upper panel), a decrease in the sfGFP signal and increasing fluorescent conversion of a caspase-3/7 substrate present in the cultures indicated specific lysis of EC-CKP organoids (Figure 4C, lower panel). Furthermore, disintegration of the EC-CKP organoid structure starting at the outer margin suggested progressive lysis of the target cells to occur layer by layer. For quantitative analysis, the relative signal changes at each time point of the experiment were determined for a larger number of individual organoids, allowing to assess average lysis of the measured organoids by the decrease in sfGFP signal. Over the 12 h observation period, growth of ErbB2-negative C-CKP organoids was inhibited to some extent by both NK-92/5.28.z and parental NK-92 cells in comparison with untreated samples, likely reflecting the limited natural cytotoxicity of the NK cells (Figure 4D, left panel). In contrast, in the case of ErbB2-expressing EC-CKP organoids a marked and progressive decrease in reporter signal indicated effective lysis over time by the CAR-NK cells, while parental NK-92 cells only had a limited growth inhibitory effect similar to that against C-CKP organoids (Figure 4D, right panel). Thereby, the extent of EC-CKP lysis by NK-92/5.28.z cells correlated with organoid size, with small organoids almost completely eliminated after 12 h, while larger organoids exhibited only partial destruction at the same time point (Figure S5).

Taken together, these data underscore the potential of the CAR-NK cells to effectively eliminate the ErbB2-expressing and malignantly transformed target cells also when growing as more complex 3D organoid structures, with extent and kinetics of cell killing dependent on exposure time and organoid size.

In vivo tumor formation of *ex vivo* generated EC-CKP mammary organoids

To investigate the potential of the *ex vivo* generated organoids to induce tumor formation *in vivo*, dissociated EC-CKP organoids were transplanted into the flanks of NSG mice, indeed resulting in the development of subcutaneous tumors (Figure 5A). For further analysis, tumor tissues were harvested at approximately 4 weeks post transplantation and processed into organoids. From these cultures, cancer cells were enriched by flow cytometric cell sorting and subjected to a second round of *in vivo* passaging, which again was followed by collection of tumor tissues and *ex vivo* culture. Tumor organoids from the first *in vivo* passage predominantly expressed EpCAM, whereas in the second *in vivo* passage a sizable population of cancer cells with reduced or absent EpCAM expression emerged (Figure S6A), indicating plasticity of the cells and progressive carcinogenesis. For further testing, $EpCAM^{+}$ and $EpCAM^{-/low}$ cells from the second *in vivo* passage of EC-CKP organoids were separated by flow cytometric cell sorting (Figure 5B). Subsequent microscopic analysis revealed marked phenotypic differences between these two cell populations, both when grown as 2D or 3D cultures (Figure 5C). While tumor-derived $EpCAM^{+}$ cells formed organoids with a morphology comparable with that of the non-passaged parental line, $EpCAM^{-/low}$ organoids exhibited more extensive branching. Furthermore, when cultured as monolayers, $EpCAM^{-/low}$ cells displayed a mesenchymal phenotype with spindle-shaped morphology and increased cell protrusions.

These morphological changes together with the loss of adhesion molecules such as EpCAM are suggestive of epithelial-to-mesenchymal transition (EMT). Hence, in a more detailed analysis we examined monolayer cultures of $EpCAM^{+}$ and $EpCAM^{-/low}$ cells from the second *in vivo* passage for expression of typical EMT markers (Figure S6B). Thereby, $EpCAM^{-/low}$ cells showed a decrease in E-cadherin, but increased expression of the mesenchymal markers N-cadherin and vimentin, as well as expression of the transcription factor zinc finger E-box-binding homeobox 1 (ZEB1), a driver of EMT.[29] Furthermore, flow cytometric analysis identified a population within the $EpCAM^{-/low}$ cells characterized by high CD44 and low or absent CD24 expression (Figure 5D), indicating the presence of cancer stem cells (CSCs). Indeed, separate transplantation of $EpCAM^{+}$ and $EpCAM^{-/low}$ cells from *in vivo* passage 2 into NSG mice revealed more rapid tumor formation and much more aggressive growth of the latter (Figure 5E), further confirming the presence of tumor-initiating cells predominantly in the $EpCAM^{-/low}$ population. Importantly both, $EpCAM^{+}$ and $EpCAM^{-/low}$ organoids derived from tumors of the third *in vivo* passage retained high ErbB2 levels (Figure S7).

Thus, while not affecting ErbB2 expression, serial *in vivo* passaging of EC-CKP organoids promoted intratumoral heterogeneity, with phenotypic and molecular changes associated with progressive malignancy persisting in subsequent organoid cultures.

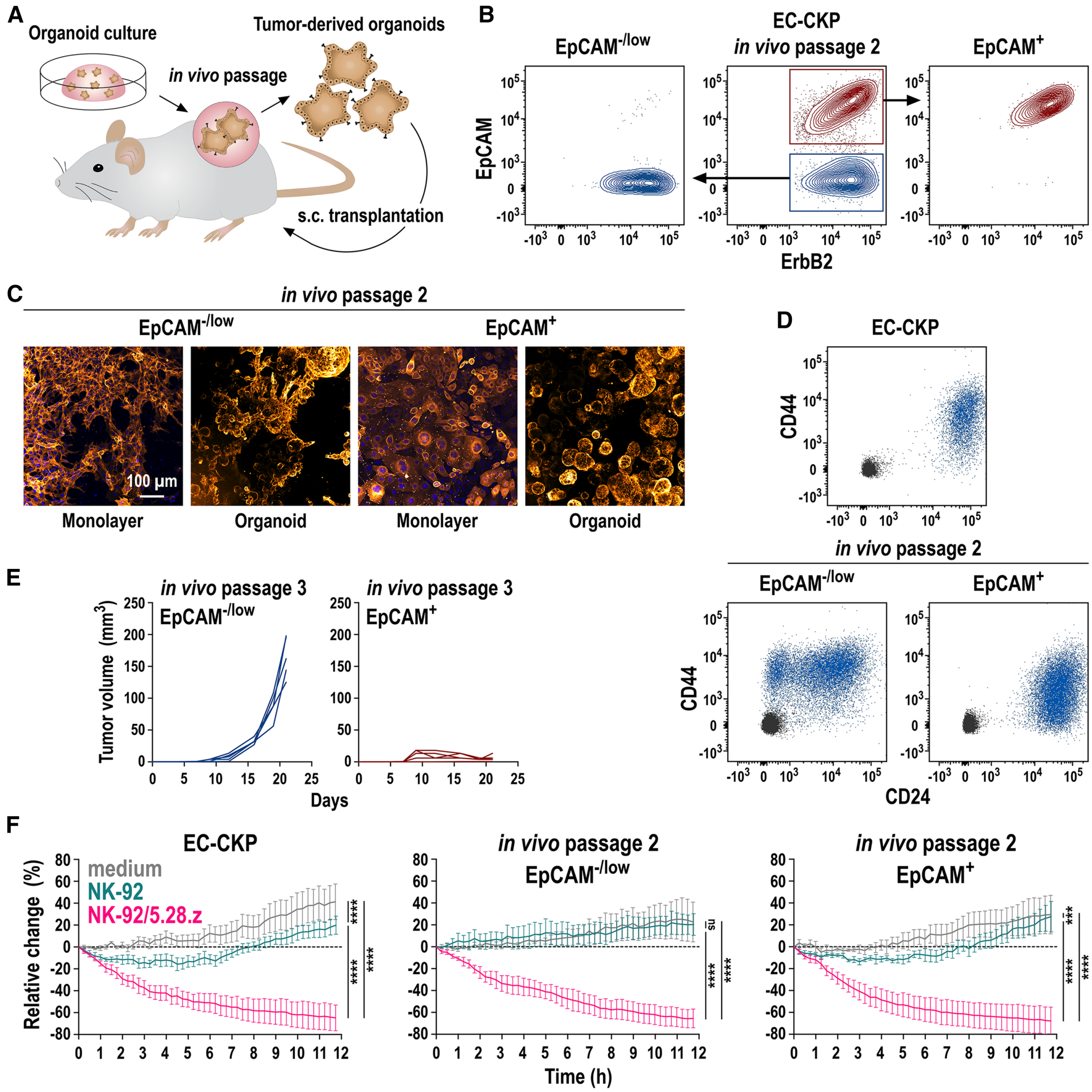

Figure 5. Phenotypic changes of tumor organoids after serial *in vivo* passaging and sensitivity to CAR-NK cell treatment

(A) Schematic overview of serial *in vivo* passaging of mammary organoids. Single-cell suspensions derived from EC-CKP organoids (1×10^6 cells) were subcutaneously transplanted into the flanks of NSG mice. Engrafted tumors were excised after 21–30 days, dissociated and expanded in Matrigel. Then single-cell suspensions were derived, tumor cells were enriched by flow cytometric cell sorting with an ErbB2-specific antibody and subjected to another round of *in vivo* passaging. (B) Tumor organoids established from the second *in vivo* passage were processed into single-cell suspensions and EpCAM$^{-/low}$ (blue) and EpCAM^{+} (red) epithelial cells were separated by flow cytometric cell sorting. (C) Morphological differences between EpCAM$^{-/low}$ and EpCAM^{+} cells from the second *in vivo* passage were assessed by immunofluorescence microscopy. Shown are maximum intensity projections of tumor cells in 2D or 3D culture, stained with a fluorochrome-conjugated anti-ErbB2 antibody (orange) and counterstained with Hoechst dye (blue). Scale bar, 100 μm. (D) Expression of CD24 and CD44 by organoid cells from the second *in vivo* passage was analyzed by flow cytometry with fluorochrome-conjugated antibodies in comparison with parental EC-CKP cells from continuous *in vitro* culture. (E) Sorted EpCAM$^{-/low}$ and EpCAM^{+} EC-CKP organoid cells (1×10^6) from the second *in vivo* passage were subcutaneously transplanted into NSG mice and tumor development was followed by caliper measurements. Tumor growth kinetics of individual animals are shown ($n = 5$ per group). (F) EpCAM$^{-/low}$ and EpCAM^{+} EC-CKP organoids from the second *in vivo* passage were co-incubated with NK-92/5.28.z or parental NK-92 cells. Kinetics of NK-mediated lysis were then determined as described for Figure 4D. EC-CKP organoids from continuous *in vitro* culture were included for comparison. Organoids kept without NK cells served as controls (medium). Mean values ± SEM from four independent experiments are shown, each analyzing three to eight organoids per condition. ****$p < 0.0001$, ***$p < 0.001$, ns, $p \geq 0.05$.

Preservation of CAR-NK cell activity against *in vivo* passaged mammary tumor cells

Considering potential treatment resistance, we examined sensitivity of organoids derived from sorted $EpCAM^{+}$ and $EpCAM^{-/low}$ cells from the second *in vivo* passage to the cytolytic activity of ErbB2-specific NK-92/5.28.z cells in microscopy-based cytotoxicity assays. As demonstrated before, non-passaged EC-CKP organoids included for comparison were effectively lysed by the CAR-NK cells, with limited growth-inhibiting activity of parental NK-92, attributable to natural cytotoxicity of the NK cells (Figure 5F, left panel). The same was found for *in vivo* passaged $EpCAM^{+}$ organoids (Figure 5F, right panel). In contrast, growth of $EpCAM^{-/low}$ organoids was no longer inhibited by parental NK-92 cells (Figure 5F, middle panel), which coincided with increased resistance to 5-fluorouracil treatment and radiation (Figure S8). But despite the more aggressive EMT and CSC phenotype of $EpCAM^{-/low}$ organoids and their resistance to standard therapies, CAR-engineered NK-92/5.28.z cells fully maintained highly effective and cancer cell-specific cytotoxicity (Figure 5F, right panel). Likewise, exposure to *in vivo* passaged $EpCAM^{-/low}$ organoids resulted in upregulation of the activation marker CD69 and cytokine production by the CAR-NK cells comparable with that observed after contact with *in vivo* passaged $EpCAM^{+}$ organoids and EC-CKP organoids from the initial *in vitro* culture (Figure S3).

Although organoid models can closely approximate patient tumors by maintaining cellular heterogeneity and architecture, *in vivo* studies provide additional benefits such as a more complex tumor microenvironment and interactions with stromal cells. Hence, $EpCAM^{-/low}$ EC-CKP organoids from the second *in vivo* passage, which best represent the heterogeneity of advanced disease, were chosen to investigate antitumoral activity of ErbB2-specific NK-92/5.28.z cells in a subcutaneous mouse tumor model. For this, 1×10^6 cancer cells from $EpCAM^{-/low}$ organoids were injected into the flanks of NSG mice. After establishment of palpable tumors, the animals were treated by peritumoral administration of 1×10^7 NK-92/5.28.z or parental NK-92 cells on days 6, 9, 12, and 15 (Figure 6A). A control group was treated with injection medium. Growth of the individual tumors was followed by caliper measurements. Two days after the last treatment, mice were sacrificed, tumors were excised, and tumor weights were determined. While rapid tumor growth was found in medium-treated animals (Figure 6B, left panel), growth kinetics were delayed to some extent in mice that received parental NK-92 cells (Figure 6B, middle panel). However, much more pronounced inhibition of tumor growth was observed in animals treated with CAR-engineered NK-92/5.28.z cells (Figure 6B, right panel), with this difference in treatment outcome also reflected by a much lower tumor weight at endpoint analysis (Figure 6C). Histological analysis of tumors collected 2 days after the last CAR-NK cell injection revealed that ErbB2 expression was retained across all treatment groups (Figure 6D). Notably, NK cell infiltration into the tumor tissue was only found in animals treated with NK-92/5.28.z cells, but not in mice that had received parental NK-92. Thereby, CAR-NK cells within the tumor were predominantly localized in close proximity to the tumor margin.

These results underscore the potent and specific *in vitro* and *in vivo* efficacy of ErbB2-specific CAR-NK cells even against highly aggressive breast cancer cells that recapitulate features of advanced disease.

DISCUSSION

In this study we investigated the antitumor activity of NK-92/5.28.z CAR-NK cells in an organoid model of ErbB2-positive breast cancer generated by activating disease-related oncogenic driver mutations in initially healthy mammary epithelial cells. This allowed us to evaluate the efficacy of the CAR-NK cells against tumor cells at an early stage of malignant transformation and against tumor cell variants exhibiting increased aggressiveness following consecutive rounds of *in vivo* passaging. In general, NK cells play a critical role in the early control of malignancies by recognizing and rapidly engaging transformed cells that downregulated self-MHC class I molecules and/or upregulated stress-induced ligands of activating NK cell receptors such as NKG2D and natural cytotoxicity receptors.[7] NK cells that express CD16 (FcγRIIIa) are also the main mediators of antibody-dependent cell-mediated cytotoxicity, which in the case of ErbB2-positive breast cancer is highly relevant for the therapeutic activity of trastuzumab.[30] Linking antibody-mediated recognition of ErbB2 directly with the transduction of strong activating signals as mediated by the CAR of NK-92/5.28.z cells has the potential to trigger more effective target cell killing than CD16 activation, as previously demonstrated in tissue culture models comparing NK-92/5.28.z with CD16-expressing NK-92 cells in combination with trastuzumab.[20,21] Furthermore, targeted cytotoxicity of NK-92/5.28.z cells was not only observed against trastuzumab-sensitive but also trastuzumab-resistant breast cancer cell lines that expressed elevated levels of ErbB2.[10]

To assess the therapeutic potential of the ErbB2-specific CAR-NK cells in a more complex and tissue-like 3D culture system, here we developed a murine organoid model of ErbB2-positive breast cancer that more accurately recapitulates tumor biology and cancer progression than conventional 2D cultures. Thereby, cultivation of single-cell suspensions from dissociated mammary fat pads of CKP mice in a basement membrane-like hydrogel promoted self-enrichment of epithelial cells and formation of complex 3D structures. These exhibited organotypic compartmentalization with a multilayered epithelium and a cytokeratin expression pattern very similar to that of intact mammary glands,[31,32] and included both basal cells and distinct luminal epithelial cell lineages.[26,33–36] ErbB2 expression and Cre-mediated induction of oncogenic driver mutations in CKP cells resulted in EC-CKP organoids that mirror key molecular alterations of ErbB2-positive breast cancer, including constitutive activation of the MAP kinase pathway,[37,38] and inactivation of p53, one of the most common mutations in breast cancer.[39] This was accompanied by an accelerated proliferation rate and a marked increase in DNA damage. EC-CKP organoids also displayed significant morphological changes such as enlarged and irregular nuclei, increased cell size, and

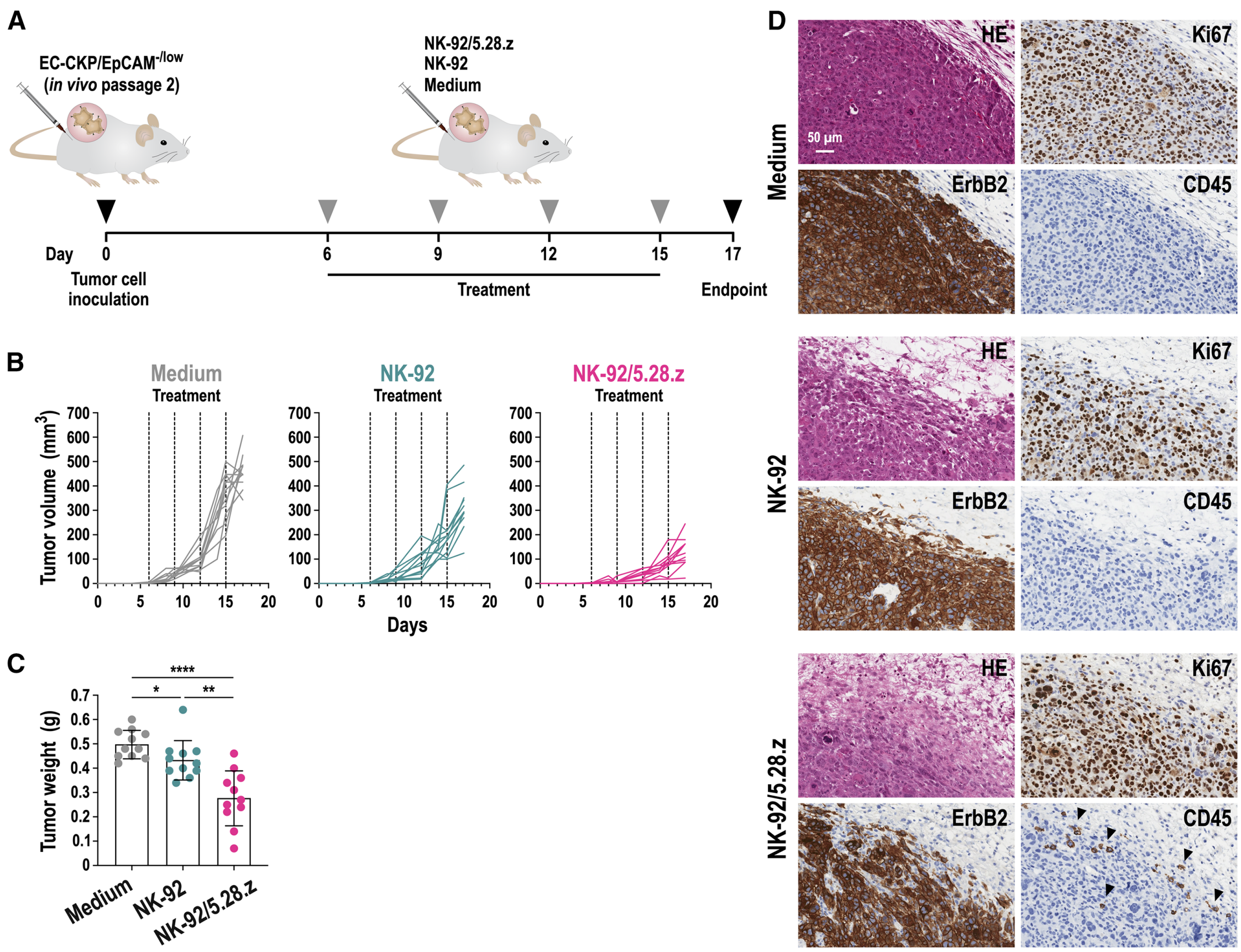

Figure 6. *In vivo* activity of NK-92/5.28.z cells against aggressive EC-CKP tumors

(A) EC-CKP/EpCAM$^{-/low}$ organoid cells from the second *in vivo* passage (1×10^6) were injected subcutaneously into the flanks of NSG mice. On days 6, 9, 12, and 15 post injection, mice were treated by peritumoral injection of 1×10^7 NK-92/5.28.z or parental NK-92 cells. Animals treated with injection medium served as control. On day 17, mice were sacrificed, tumors were harvested and weighed. (B) Development of subcutaneous EC-CKP/EpCAM$^{-/low}$ tumors was followed by caliper measurements. Tumor growth kinetics for the individual animals from each treatment group are shown. (C) Tumor weights at endpoint analysis on day 17. Mean values ± SD are shown; $n = 11$ per treatment group. ****$p < 0.0001$, **$p < 0.01$, *$p < 0.05$. (D) To examine tumor architecture, proliferative activity, ErbB2 expression and NK cell infiltration, consecutive slides of tumors collected on day 17 were stained with hematoxylin and eosin (HE), or analyzed by immunohistochemistry with antibodies specific for Ki67, ErbB2, and human CD45. Shown are microscopic images of a representative tumor from each treatment group taken at 20× magnification. Infiltrating CD45-positive NK-92/5.28.z cells are indicated by arrowheads. Scale bar, 50 µm.

abnormal tissue architecture, which are characteristic for malignant cells.[40,41] While expression of mutated Kras as used in our model is found in less than 2% of human breast cancers,[42] it reliably induces downstream alterations such as constitutive MAP kinase signaling typical for ErbB2-positive disease. This allowed us to compare ErbB2-expressing EC-CKP and ErbB2-negative C-CKP organoids, with both cell types displaying a similar degree of malignant transformation irrespective of their ErbB2 expression levels (see Figure S4).

When tested with dissociated EC-CKP organoids in 2D culture, NK-92/5.28.z cells exhibited specific CAR-mediated activation and degranulation demonstrated by CD107a expression and granule polarization, and displayed targeted cytotoxicity against the transformed cells, while ErbB2-negative CKP cells did not induce such effects. This aligns with our previous findings that demonstrated selective elimination of ErbB2-positive cancer cells of different solid tumor origins by NK-92/5.28.z cells.[10,15,17,43] While the CAR-NK cells retained specific cytotoxicity also against intact EC-CKP organoids, their efficacy in short-term assays was less pronounced than against dissociated target cells. This can be attributed to the increased structural complexity of the 3D model, which could reduce NK cell motility, influence cellular interactions, and increase apoptosis resistance of target cells as demonstrated in other 3D culture systems.[44–47] Our microscopic studies revealed that NK-92/5.28.z cells did not infiltrate deeply into EC-CKP organoids, but accumulated primarily at the periphery, from where they lysed the breast cancer cells by progressing layer by layer. Accordingly, the extent of lysis of individual organoids during the observation period was also dependent on organoid size, which is in agreement with target cells in the inner core only becoming accessible upon destruction of the outer cell layers. Limited infiltration has also been described for tumor tissues, where NK cells are typically restricted to peripheral

regions, perivascular areas within the tumor, and tertiary lymphoid structures.[48–51] Still, upon prolonged interaction effective lysis of EC-CKP organoids by NK-92/5.28.z cells was observed, with CAR-independent natural cytotoxicity likely contributing to target cell killing at high E/T cell ratios as indicated by the limited activity of the CAR-NK cells against malignantly transformed but ErbB2-negative C-CKP organoids.

Serial *in vivo* passaging of EC-CKP organoids revealed considerable plasticity of the tumor cells, demonstrated by the emergence of cells with an EMT phenotype. While retaining stable ErbB2 expression, respective organoid derivatives exhibited notable phenotypic alterations, including elevated expression of mesenchymal markers, decreased expression of epithelial markers, the presence of a CSC subset, and markedly enhanced tumor initiating potential, collectively indicating a hybrid epithelial/mesenchymal state.[52–54] This transition, which typically occurs during tumor progression, can contribute to increased resistance to both conventional treatments and immunotherapies.[55–57] Indeed, *in vivo* passaged EpCAM$^{-/low}$ EC-CKP organoids in contrast to EpCAM^{+} and non-passaged EC-CKP cells showed increased resistance to 5-fluorouracil treatment and radiation, and were no longer sensitive to the natural cytotoxicity of parental NK-92 cells. In sharp contrast, CAR-engineered NK-92/5.28.z cells were not affected by this EMT-associated treatment resistance, and fully retained high and specific activity against organoids established from the more aggressive EpCAM$^{-/low}$ EC-CKP tumor cells. Importantly, pronounced therapeutic activity of NK-92/5.28.z against the EpCAM$^{-/low}$ breast cancer cells was maintained in a subcutaneous *in vivo* tumor model that mimicked advanced disease, corroborating the findings from the *in vitro* organoid model. Thereby, CAR-engineered NK-92/5.28.z cells were still found in the tumors 2 days after treatment, demonstrating their ability to infiltrate at least to some extent into the tumor tissue. This was not the case for parental NK-92 cells lacking the ErbB2-specific CAR. Future work will now be directed at evaluating the activity of NK-92/5.28.z cells against EC-CKP tumors in orthotopic and metastasizing breast carcinoma models.

In conclusion, our data underscore the high potential of NK-92/5.28.z for the therapy of ErbB2-positive breast cancer, where these off-the-shelf CAR-NK cells may complement treatment options for advanced disease resistant to tyrosine kinase inhibitors, monoclonal antibodies, and other current ErbB2-targeted modalities. Feasibility and safety of administering NK-92/5.28.z cells have already been demonstrated in the dose escalation part of our ongoing phase I clinical trial in glioblastoma, with signs of treatment-induced immune activation observed in some of the patients.[19] This can be expected to aid rapid clinical translation of NK-92/5.28.z cells also for the treatment of breast cancer and other ErbB2-positive malignancies of solid tumor origin.

MATERIALS AND METHODS

Cells and culture conditions

NK-92 cells[58] (kindly provided by NantKwest, Culver City, CA) and the CAR-engineered cell line NK-92/5.28.z[15] were grown in X-VIVO 10 medium (Lonza, Cologne, Germany) supplemented with 5% heat-inactivated human AB plasma (German Red Cross Blood Donation Service Baden-Württemberg-Hessen, Frankfurt, Germany) and 100 IU/mL IL-2 (Proleukin; Novartis, Basel, Switzerland). HEK293T cells (ATCC, Manassas, VA) were cultured in DMEM (Gibco, Thermo Fisher Scientific, Darmstadt, Germany) supplemented with 10% heat-inactivated FBS (Capricorn Scientific, Ebsdorfergrund, Germany), 2 mM L-glutamine, 100 U/mL penicillin, and 100 μg/mL streptomycin (all Gibco).

Generation and propagation of murine mammary organoids

Organoids were generated from mammary gland tissue of CC10-CreERT2 *Kras*$^{LSLG12Vgeo/WT}$ *Tp53*$^{fl/fl}$ (CKP) mice.[25] The fourth mammary fat pads from 6- to 12-week-old female mice were harvested, lymph nodes were removed, tissues were minced, and enzymatically dissociated in Advanced DMEM/F12 medium (Gibco) supplemented with 50 U/mL collagenase (Sigma-Aldrich, Merck, Darmstadt, Germany) under orbital shaking (130 rpm) at 37°C. After 45–60 min, 20 U/mL DNAse (Sigma-Aldrich) was added, and incubation was continued for another 20 min. Dissociated cells were collected by centrifugation, washed, and either kept in organoid growth medium (Advanced DMEM/F12 containing 5% heat-inactivated FBS, 2 mM L-glutamine, 100 U/mL penicillin, 100 μg/mL streptomycin, 5 μg/mL insulin [Sigma-Aldrich], 10 ng/mL epidermal growth factor [EGF], and 20 ng/mL fibroblast growth factor [both PeproTech, Hamburg, Germany]) or directly resuspended in 200–300 μL growth factor reduced Matrigel (Corning, New York, NY) per fat pad, and seeded in 24-well plates (40–50 μL/well). Initial cultures (< passage 2) were maintained in the presence of 10 μM Rho-kinase inhibitor Y-27632 (Selleckchem, Houston, TX).

Epithelial cell enrichment was achieved by serial passaging. Once or twice weekly, Matrigel was partially dissolved in ice-cold DPBS (Gibco) containing 1 U/mL Dispase II (Roche Diagnostics, Mannheim, Germany). After incubation for 5–10 min at 37°C, the partially digested cell/Matrigel mixture was pelleted by centrifugation, resuspended in 1 mL TrypLE Express (Thermo Fisher Scientific) and incubated for another 10–15 min at 37°C, with enzymatic dissociation supported by pipetting the cell/Matrigel mixture up and down every 5 min. Dissociated cells were washed with growth medium, resuspended in ice-cold Matrigel, seeded in 24-well plates, and overlaid with growth medium after solidifying. To follow epithelial cell enrichment, primary cells and cells from each passage were isolated from Matrigel and examined by flow cytometry. Single-cell suspensions were washed, treated with Fc blocking solution (TruStain FcX; BioLegend, Koblenz, Germany), and incubated with PE-conjugated antibodies specific for lineage markers CD31 (30–90), CD45 (30-F11), and Ter-119 (Ter-119), and a BV421-conjugated anti-EpCAM antibody (G8.8) (all BioLegend). Subsequently, cells were stained with Fixable Viability Dye eFluor 780 (Thermo Fisher Scientific), and percentages of Lineage^{-}, Lineage^{+}, and EpCAM^{+} cells among viable cells were determined using a FACSCanto II flow cytometer (BD Biosciences, Heidelberg, Germany).

Secondary tumor organoids were established through serial *in vivo* passaging of mammary epithelial cells in NSG mice. After engraftment, tumors were excised, and 3D cell cultures were generated as described for mammary gland tissue. Tumor cells from expanded cultures were enriched after enzymatic dissociation by flow cytometric cell sorting using AF647-conjugated anti-ErbB2 antibody (24D2; BioLegend). Single-cell suspensions derived from tumor organoids of each *in vivo* passage were stained with BV421-conjugated anti-EpCAM antibody and analyzed with a FACSCanto II flow cytometer. $EpCAM^{-/low}$ and $EpCAM^{+}$ cells of the second *in vivo* passage were enriched by flow-cytometric cell sorting using a FACSAria Fusion flow cytometer (BD Biosciences). Dissociated organoids from the second *in vivo* passage were stained with APC-conjugated anti-CD24 (M1/69; BioLegend) and PE-Cy7-conjugated anti-CD44 antibodies (IM7; BioLegend), and analyzed using a FACSCanto II flow cytometer.

Analysis of organoid architecture and cellular composition

To investigate cytokeratin expression in intact 3D structures, single-cell suspensions derived from organoids were resuspended in Matrigel and seeded into 96-well plates. After a few days, organoids in Matrigel were fixed for 60 min in DPBS containing 2% paraformaldehyde (PFA) and 0.25% glutaraldehyde (both Carl Roth, Karlsruhe, Germany), washed with DPBS, and incubated for 10 min at 37°C in 0.05% trypsin solution for antigen retrieval. Fixed organoids were stained overnight with anti-cytokeratin 8 + 18 (polyclonal) and anti-cytokeratin 14 (EPR17350) antibodies (both Abcam, Cambridge, UK), followed by species-specific AF488- and AF647-conjugated secondary antibodies (both Abcam) for 1–2 h, before sample analysis using a CQ1 Confocal Quantitative Image Cytometer (Yokogawa, Tokyo, Japan). To evaluate the cellular composition of organoid cultures by flow cytometry, single-cell suspensions were derived from organoids, stained with APC-conjugated anti-CD24 (M1/69), Pacific blue-conjugated anti-CD29 (HMβ1-1), PerCP-Cy5.5-conjugated anti-CD61 (2C9.G2), PE-conjugated anti-CD133 (315-2C11), APC-Cy7-conjugated anti-c-Kit (2B8), FITC-conjugated anti-EpCAM (G8.8), and PE-Cy7-conjugated anti-Sca-1 (D7) antibodies (all from BioLegend), and analyzed using a FACSCanto II flow cytometer.

Generation of genetically modified organoid lines

Genetic modification of mammary organoids from CKP mice was performed using ecotropic MLV-pseudotyped viral vectors. For expression of Cre recombinase, lentiviral transfer plasmid pRRLSIN.cPPT.PGK-Cre.WPRE was used, which is based on the pRRLSIN.cPPT.PGK-GFP.WPRE vector backbone (plasmid no. 12252; Addgene, Watertown, MA), with Cre replacing the GFP sequence. For co-expression of ErbB2 and Cre, lentiviral transfer plasmid pS-ErbB2-Cre-W was generated by *de novo* synthesis of sequences encompassing human ErbB2 and IRES-Cre (GeneArt, Thermo Fisher Scientific), and stepwise assembly in the pHR'SIN-SIEW vector backbone,[59] thereby replacing the initial IRES-EGFP sequences of the vector. Lentiviral particles were produced by co-transfecting HEK293T cells with the respective lentiviral transfer plasmid together with packaging and envelope plasmids pCMVΔR8.91[60] and pEF1α-envMoMLV (M187)[61] using FuGENE HD Transfection Reagent (Promega, Walldorf, Germany) according to the manufacturer's recommendations. Two to 3 days post transfection, virus-containing supernatants were collected. CKP organoids were isolated from Matrigel, fully dissociated, and resuspended in 2 mL of virus-containing supernatant in a 6-well plate in the presence of 8 μg/mL polybrene (Sigma-Aldrich). Following a 90 min spinfection (1,800 rpm, 32°C), the cells were incubated for an additional 4.5 h at 37°C, washed, and re-seeded in Matrigel.

Cre-expressing organoids (C-CKP) were selected based on Cre-induced *Tp53* deletion using the MDM2 inhibitor Nutlin-3a (10 μM) (Selleckchem). For enrichment of ErbB2-expressing organoid cells (EC-CKP), organoids were isolated from Matrigel, fully dissociated, stained with AF647-conjugated ErbB2-specific antibody (24D2; BioLegend), and sorted using a FACSAria Fusion flow cytometer. For analysis of ErbB2 surface expression on intact EC-CKP organoids in Matrigel, organoids were stained with AF647-labeled ErbB2-specific antibody, fixed in DPBS containing 2% PFA and 0.25% glutaraldehyde, counterstained with Hoechst 33342 (Thermo Fisher Scientific), and imaged using a CQ1 Confocal Quantitative Image Cytometer. Mammary epithelial cells and organoids expressing firefly luciferase (Luc2) and superfolder green fluorescent protein (sfGFP) were generated by transduction with lentiviral particles generated as described above using transfer plasmid pS-luc-V5-T2A-sfGFP-W (kindly provided by Christian Buchholz, Paul-Ehrlich-Institut, Langen, Germany). sfGFP-expressing cells were enriched by flow-cytometric cell sorting with a FACSAria Fusion flow cytometer.

Analysis of p53 status

Tp53 status was analyzed by PCR of genomic DNA as previously described,[62] using primers $Tp53^{D2.10}$1F (5′-CACAAAAACAGGT TAAACCCAG-3′) and $Tp53^{F2.10}$1R (5′-AGCACATAGGAGG CAGAGAC-3′), and primers $Tp53^{D2.10}$1F and $Tp53^{D2.10}$10R (5′-GAAGACAGAAAAGGGGAGGG-3′) to identify $Tp53^{F2\text{-}10}$ and recombined $Tp53^{\Delta 2\text{-}10}$ alleles, respectively. To confirm absence of p53 protein expression in EC-CKP organoids, single-cell suspensions were derived, fixed, and permeabilized using the Foxp3/Transcription Factor Staining Buffer Set (Thermo Fisher Scientific), and then stained overnight with anti-p53 antibody (1C12; Cell Signaling Technology, Leiden, the Netherlands), followed by incubation with species-specific AF647-conjugated secondary antibody (Invitrogen, Thermo Fisher Scientific) and flow cytometric analysis. CKP organoids served as control. Similarly, p53 expression by EC-CKP and C-CKP organoids in comparison with CKP organoids was investigated by immunofluorescence microscopy. Single cells from dissociated organoids were seeded as 2D cultures in chamber slides coated with poly-L-lysine (Sigma-Aldrich), fixed and permeabilized using the Foxp3/Transcription Factor Staining Buffer Set, and incubated for 30–60 min in blocking solution (3% bovine serum albumin in permeabilization buffer). Then, cells were incubated overnight at 4°C with anti-p53 antibody (1C12), followed by staining

with species-specific AF647-conjugated secondary antibody (Invitrogen, Thermo Fisher Scientific) and counterstaining with Hoechst 33342. Images were acquired with a CQ1 Confocal Quantitative Image Cytometer and analyzed using ImageJ software as described below.

Analysis of Kras^{G12V} expression and activity

Expression of Kras^{G12Vgeo} was confirmed by detecting enzymatic activity of the linked β-Geo reporter. Cells in 2D or 3D cultures were fixed in DPBS containing 2% PFA and 0.2% glutaraldehyde, washed with DPBS and equilibration buffer (2 mM magnesium chloride, 5 mM potassium ferrocyanide, 5 mM potassium ferricyanide in DPBS [pH 7.4]; Carl Roth, Karlsruhe, Germany), and incubated overnight in X-Gal staining solution (equilibration buffer with 1 mg/mL X-Gal [5-bromo-4-chloro-3-indolyl-β-D-galactopyranoside]; Carl Roth). X-Gal staining was documented using an Axiovert 25 microscope (Zeiss, Oberkochen, Germany). Kras^{G12V}-mediated activation of MAPK signaling was assessed by immunoblot analysis. Total proteins of whole-cell lysates (25 μg per lane) were separated by SDS-PAGE under reducing conditions, transferred to PVDF membranes, and probed with antibodies specific for phospho-Erk1/2 (E10) and phospho-c-Raf (Ser259) (Cell Signaling Technology), followed by HRP-conjugated species-specific secondary antibodies and chemiluminescent detection. Antibodies detecting total Erk1/2 (polyclonal; Cell Signaling Technology) and γ-tubulin (polyclonal; Sigma-Aldrich) were used as controls. For quantification of phospho-Erk1/2 by flow cytometry, single-cell suspensions were fixed, permeabilized, and stained overnight with phospho-Erk1/2-specific antibody, followed by incubation for 1 h with species-specific fluorochrome-conjugated secondary antibody. Data were analyzed using FlowJo software (version 10.9.0; BD Biosciences).

Detection of DNA double-strand breaks

DNA double-strand breaks were identified by immunofluorescence microscopy detecting γ-H2AX. Dissociated organoid cells were cultured in chamber slides for 1–2 days, stained with BV421-conjugated anti-EpCAM antibody (G8.8) to visualize cell bodies, fixed with 1% PFA for 10 min, permeabilized with 0.5% Triton X-100 in DPBS for 15 min, and blocked for 1 h with 5% FBS in DPBS. AF488-conjugated anti-γ-H2AX antibody (Ser139; BioLegend) was added, and cells were incubated overnight at 4°C, followed by staining the nuclei with propidium iodide (PI) solution (1 μg/mL). Imaging was performed with a CQ1 Confocal Quantitative Image Cytometer at 60× magnification using 405, 488, and 561 nm lasers.

Immunohistochemistry of organoid and tumor sections

Intact organoids were isolated from Matrigel, fixed in 2% PFA for 4 h, washed, briefly stained with hematoxylin, transferred to HistoGel (Thermo Fisher Scientific), and incubated on ice for 20 min. Tumors from the treatment experiment described below were removed 2 days after the final injection of NK cells and fixed in 4% PFA. Fixed organoid or tumor samples were dehydrated using an ASP300S device (Leica, Wetzlar, Germany), embedded in paraffin, and sectioned (3–4 μm). Sections were deparaffinized and automatically processed using a Bond-Max device (Leica) and the Bond Polymer Refine Detection system (Leica) according to the manufacturer's recommendations. Organoid sections were stained with Ki67-specific antibody (D3B5; Cell Signaling Technology). Tumor sections were stained with Ki67-specific antibody (D3B5), human CD45-specific antibody (EP322Y; Abcam), or ErbB2-specific antibody (polyclonal; Dako, Agilent Technologies, Waldbronn, Germany). All sections were then incubated with HRP-conjugated species-specific secondary antibodies, followed by detection with HRP substrate 3,3′-diaminobenzidine tetrahydrochloride hydrate (DAB) (Leica) and counterstaining with hematoxylin. Stained organoid sections were imaged with an Axio Imager 2 microscope (Zeiss), followed by image analysis using QuPath software as described below. Tumor sections were scanned on a ScanScope CS2 scanner and analyzed using Aperio eSlide Manager software (both Leica).

Degranulation of NK cells

Degranulation of NK cells was assessed by analyzing surface exposure of CD107a (LAMP-1). NK cells were incubated in 96-well plates at 2×10^5 cells/well together with an equal number of target cells in the presence of PE-conjugated anti-CD107a antibody (BioLegend) and Golgi-Stop (BD Biosciences). NK cells kept in the absence of target cells and NK cells stimulated with 5 ng/mL PMA and 50 ng/mL ionomycin (both Sigma-Aldrich) served as controls. After 4 h of incubation at 37°C, the cells were washed with DPBS, stained with BV421-labeled anti-CD56 antibody (NCAM16.2; BD Biosciences), and the proportion of CD107a-positive NK cells was determined by flow cytometry.

For microscopic analysis of cytotoxic granule exocytosis, dissociated organoid cells modified with the Luc2-sfGFP reporter construct were seeded in chamber slides coated with fibronectin (Sigma-Aldrich) 2–3 h prior to the addition of NK-92 or NK-92/5.28.z cells labeled with LysoTracker Deep Red (Thermo Fisher Scientific). BioTracker NucView 405 Blue Caspase-3 Dye (Merck Millipore, Darmstadt, Germany) was added to visualize caspase activation. Cells were followed for 5 h, with phase-contrast and fluorescent images acquired every 15 min at 60× magnification with a CQ1 Confocal Quantitative Image Cytometer using 405, 488, and 561 nm lasers. Images were processed as maximum intensity projections (MIPs) and analyzed with ImageJ software as described below.

CD69 and cytokine expression by NK cells

For analysis of CD69 expression, NK cells and target cells were incubated at an E/T ratio of 10:1. After 4 h, cells were washed, stained with PE-labeled anti-CD56 antibody (HCD56; BioLegend) and BV421-labeled anti-CD69 antibody (FN50; BD Biosciences) and analyzed by flow cytometry. To assess cytokine expression, NK cells were incubated with target cells at an E/T ratio of 1:1. After 1 h, Golgi-Stop and Golgi-Plug (BD Biosciences) were added according to the manufacturer's recommendations. Then cells were incubated for another 3 h, washed and stained with BV421-labeled anti-CD56 antibody (NCAM16.2; BD Biosciences) prior to fixation and intracellular staining using the Foxp3/Transcription Factor Staining

Buffer Set. In brief, cells were fixed for 30 min at room temperature, washed with permeabilization buffer, and incubated with FITC-conjugated anti-INF-γ (B27; BioLegend) and PE-conjugated anti-TNF-α (Mab11; eBioscience, Thermo Fisher Scientific) antibodies. After 40 min at room temperature, cells were washed twice with permeabilization buffer and analyzed by flow cytometry.

Cytotoxicity assays

Cytolytic activity of NK cells against dissociated organoids was assessed using flow cytometry-based assays basically as described.[15] Organoids were isolated from Matrigel, dissociated as described above, and stained with Calcein Violet AM (CV) (CellTrace; Invitrogen, Thermo Fisher Scientific) according to the manufacturer's recommendations. Then, dissociated organoid cells were co-incubated with NK-92/5.28.z or parental NK-92 cells at different E/T ratios for 4 h at 37°C, followed by staining with PI. Dead target cells were identified as CV/PI double-positive and quantified by flow cytometry with a FACSCanto II flow cytometer or an LSRFortessa Cell Analyzer (BD Biosciences). Spontaneous target cell lysis in the absence of NK cells was subtracted to calculate specific cytotoxicity. Data were analyzed using FlowJo software.

Cytotoxicity of NK cells against intact organoids was quantified using luciferase reporter assays. Luciferase-expressing organoids were isolated from Matrigel and seeded in fibronectin-coated optical 96-well plates in growth medium. After adherence, medium was replaced with 100 μL/well X-VIVO 10 containing 150 μg/mL VivoGlo Luciferin (Promega). Organoids were incubated for 30 min, and bioluminescence was measured using a SpectraMax iD3 microplate reader (Molecular Devices, San José, CA). Absolute numbers of viable cells per well were calculated using a standard curve established with luciferase-expressing organoid cells from monolayer cultures, and NK cells were added at defined E/T ratios. Bioluminescence was measured again after the desired co-incubation period to determine the decrease in luciferase activity as a measure of target cell lysis.

For real-time cytotoxicity studies, intact organoids modified with the Luc2-sfGFP reporter construct were isolated from Matrigel, seeded on fibronectin-coated optical petri dishes or well plates, and incubated in growth medium at 37°C for 1 h before addition of NK-92 or NK-92/5.28.z cells labeled with Far Red (CellTrace; Invitrogen, Thermo Fisher Scientific). BioTracker NucView 405 Blue Caspase-3 Dye was added for real-time detection of caspase-3 and caspase-7 activity. Time-lapse microscopy was performed over a 12 h period, with phase-contrast and fluorescent images acquired every 15 min at 20× magnification with a CQ1 Confocal Quantitative Image Cytometer using 405, 488, and 640 nm lasers. Images were processed as MIPs and analyzed with ImageJ software as described below.

Image processing and analysis

Image analysis was performed with QuPath (version 0.5.1)[63] and ImageJ (version 1.54f; National Institutes of Health, Bethesda, MD; https://imagej.net/ij/). QuPath software was used to quantify Ki67-positive areas after immunohistochemical staining of organoid sections. Initially, total organoid areas were determined, and images were processed by adjusting brightness and contrast. The DAB signal was segmented (resolution: full; channel: DAB; prefilter: Gaussian; smoothing sigma: 1; threshold: 0.15), and the Ki67-positive region was measured to subsequently determine the Ki67-positive area (%) within an organoid. To ensure accurate and consistent image analysis, customized macros were implemented in ImageJ for quantification of organoid area, nucleus area, and mean fluorescence intensity (MFI) upon staining with anti-p53 antibody (see supplemental methods). For quantitative time-lapse analysis, background-subtracted MFIs from the sfGFP signals of individual organoids were determined with ImageJ at each time point. Relative changes in signals were then calculated by comparing differences at each time point with initial values. Representative MIPs for microscopy-based analysis of granule exocytosis and cytotoxicity assays were analyzed with ImageJ by processing fluorescent channels using the Find Edges application, followed by increasing contrast and brightness. Consistent values for these adjustments were applied to all images within an experiment to maintain uniformity.

Animal experiments

For *in vivo* passaging of EC-CKP cells, dissociated mammary organoids (1×10^6 cells) were injected into the right flank of 6- to 12-week-old female NOD-SCID IL-2R γ^{null} (NSG) mice. The health status of the animals was monitored regularly, and tumor size was measured using a caliper. Mice were sacrificed approximately 4 weeks post transplantation, tumors were harvested, processed into organoid cultures, and sorted for ErbB2-expressing cells as described above. *In vivo* antitumoral activity of NK-92/5.28.z cells was analyzed in a subcutaneous mammary carcinoma model. For this, 1×10^6 EpCAM$^{-/low}$ EC-CKP organoid cells from the second *in vivo* passage were injected subcutaneously into the right flank of 6-week-old female NSG mice at day 0. After tumor engraftment, animals were treated by peritumoral injection of 1×10^7 NK-92/5.28.z or parental NK-92 cells at days 6, 9, 12, and 15. Animals treated with injection medium served as control. Health status was monitored daily, and tumor volumes were determined by caliper measurements. Mice were sacrificed on day 17, tumors were harvested, and tumor weights were determined. All animal experiments were approved by the responsible government committee (Regierungspräsidium Darmstadt, Darmstadt, Germany; protocol no. F123/2005), and were conducted in the animal facility of Georg-Speyer-Haus according to all applicable guidelines and regulations.

Statistical analysis

Welch's unequal variances t test was employed for statistical analysis of differences between two groups. For correlation analysis, the Pearson correlation coefficient was calculated. Data from time-lapse experiments and tumor growth kinetics were evaluated using one-way ANOVA followed by post hoc Tukey analysis. p values <0.05 were considered statistically significant and are indicated as follows: $^{****}p < 0.0001$, $^{***}p < 0.001$, $^{**}p < 0.01$, $^{*}p < 0.05$. All statistical

analyses were performed with Prism 10 (version 10.1.1.323; GraphPad Software, Boston, MA).

DATA AVAILABILITY

Data supporting the findings of this study are available within the article, the supplemental information, or from the corresponding author upon reasonable request.

ACKNOWLEDGMENTS

We thank Petra Dinse for preparation of tissue slides and immunohistochemical staining, Annette Klemke for flow cytometric cell sorting, Barbara Uherek and Thorsten Geyer for technical assistance, and the staff at the animal facility of Georg-Speyer-Haus for their support. This work was supported in part by a grant from LOEWE Center Frankfurt Cancer Institute (FCI) (HMWK III L 5-519/03/03.001-0015) and institutional funds of Georg-Speyer-Haus. Georg-Speyer-Haus is funded jointly by the German Federal Ministry of Health and the Hessian Ministry of Science and Research, Arts and Culture.

AUTHOR CONTRIBUTIONS

J.R. and W.S.W. designed the study. J.R., A.K., I.K., M.P., M.B., and A.W. performed the experiments. J.R. and W.S.W. analyzed the data. S.S., T.A., T.T., S.R., C.Z., and E.B. provided critical input, reagents and the CKP mouse model. J.R. and W.S.W. wrote the manuscript. All authors contributed to the critical review and editing of the manuscript and approved the final version.

DECLARATION OF INTERESTS

A.K., C.Z., T.T., and W.S.W. are named as inventors on patents and patent applications in the field of CAR-NK cells and cancer immunotherapy owned by their respective academic institutions. E.B. is a co-founder and CSO of ImmuneNTech.

SUPPLEMENTAL INFORMATION

Supplemental information can be found online at https://doi.org/10.1016/j.ymthe.2025.04.033.

REFERENCES

1. Bray, F., Laversanne, M., Sung, H., Ferlay, J., Siegel, R.L., Soerjomataram, I., and Jemal, A. (2024). Global cancer statistics 2022: GLOBOCAN estimates of incidence and mortality worldwide for 36 cancers in 185 countries. CA. Cancer J. Clin. *74*, 229–263. https://doi.org/10.3322/caac.21834.

2. Hynes, N.E., and MacDonald, G. (2009). ErbB receptors and signaling pathways in cancer. Curr. Opin. Cell Biol. *21*, 177–184. https://doi.org/10.1016/j.ceb.2008.12.010.

3. Swain, S.M., Shastry, M., and Hamilton, E. (2023). Targeting HER2-positive breast cancer: advances and future directions. Nat. Rev. Drug Discov. *22*, 101–126. https://doi.org/10.1038/s41573-022-00579-0.

4. Park-Simon, T.W., Müller, V., Jackisch, C., Albert, U.S., Banys-Paluchowski, M., Bauerfeind, I., Blohmer, J.U., Budach, W., Dall, P., Ditsch, N., et al. (2023). Arbeitsgemeinschaft Gynäkologische Onkologie recommendations for the diagnosis and treatment of patients with early breast cancer: update 2023. Breast Care (Basel) *18*, 289–305. https://doi.org/10.1159/000531578.

5. Elghazaly, H., Azim, H.A., Rugo, H.S., Cameron, D., Swain, S.M., Curigliano, G., Harbeck, N., Tripathy, D., Arun, B., Aapro, M., et al. (2024). Tailoring neoadjuvant systemic therapy in breast cancer: "The advent of a personalized approach"-The Breast-Gynecological and Immuno-Oncology International Cancer Conference (BGICC) consensus and recommendations. Cancer *130*, 3251–3271. https://doi.org/10.1002/cncr.35389.

6. Duro-Sánchez, S., Alonso, M.R., and Arribas, J. (2023). Immunotherapies against HER2-Positive Breast Cancer. Cancers (Basel) *15*. https://doi.org/10.3390/cancers15041069.

7. Pahl, J., and Cerwenka, A. (2017). Tricking the balance: NK cells in anti-cancer immunity. Immunobiology *222*, 11–20. https://doi.org/10.1016/j.imbio.2015.07.012.

8. Shimasaki, N., Jain, A., and Campana, D. (2020). NK cells for cancer immunotherapy. Nat. Rev. Drug Discov. *19*, 200–218. https://doi.org/10.1038/s41573-019-0052-1.

9. Maskalenko, N.A., Zhigarev, D., and Campbell, K.S. (2022). Harnessing natural killer cells for cancer immunotherapy: dispatching the first responders. Nat. Rev. Drug Discov. *21*, 559–577. https://doi.org/10.1038/s41573-022-00413-7.

10. Zhang, C., Oberoi, P., Oelsner, S., Waldmann, A., Lindner, A., Tonn, T., and Wels, W.S. (2017). Chimeric antigen receptor-engineered NK-92 cells: An off-the-shelf cellular therapeutic for targeted elimination of cancer cells and induction of protective antitumor immunity. Front. Immunol. *8*, 533. https://doi.org/10.3389/fimmu.2017.00533.

11. Laskowski, T.J., Biederstädt, A., and Rezvani, K. (2022). Natural killer cells in antitumour adoptive cell immunotherapy. Nat. Rev. Cancer *22*, 557–575. https://doi.org/10.1038/s41568-022-00491-0.

12. Dagher, O.K., and Posey, A.D., Jr. (2023). Forks in the road for CAR T and CAR NK cell cancer therapies. Nat. Immunol. *24*, 1994–2007. https://doi.org/10.1038/s41590-023-01659-y.

13. Liu, E., Marin, D., Banerjee, P., Macapinlac, H.A., Thompson, P., Basar, R., Nassif Kerbauy, L., Overman, B., Thall, P., Kaplan, M., et al. (2020). Use of CAR-transduced natural killer cells in CD19-positive lymphoid tumors. N. Engl. J. Med. *382*, 545–553. https://doi.org/10.1056/NEJMoa1910607.

14. Marin, D., Li, Y., Basar, R., Rafei, H., Daher, M., Dou, J., Mohanty, V., Dede, M., Nieto, Y., Uprety, N., et al. (2024). Safety, efficacy and determinants of response of allogeneic CD19-specific CAR-NK cells in CD19(+) B cell tumors: a phase 1/2 trial. Nat. Med. *30*, 772–784. https://doi.org/10.1038/s41591-023-02785-8.

15. Schönfeld, K., Sahm, C., Zhang, C., Naundorf, S., Brendel, C., Odendahl, M., Nowakowska, P., Bönig, H., Köhl, U., Kloess, S., et al. (2015). Selective inhibition of tumor growth by clonal NK cells expressing an ErbB2/HER2-specific chimeric antigen receptor. Mol. Ther. *23*, 330–338. https://doi.org/10.1038/mt.2014.219.

16. Nowakowska, P., Romanski, A., Miller, N., Odendahl, M., Bönig, H., Zhang, C., Seifried, E., Wels, W.S., and Tonn, T. (2018). Clinical grade manufacturing of genetically modified, CAR-expressing NK-92 cells for the treatment of ErbB2-positive malignancies. Cancer Immunol. Immunother. *67*, 25–38. https://doi.org/10.1007/s00262-017-2055-2.

17. Zhang, C., Burger, M.C., Jennewein, L., Genßler, S., Schönfeld, K., Zeiner, P., Hattingen, E., Harter, P.N., Mittelbronn, M., Tonn, T., et al. (2016). ErbB2/HER2-specific NK cells for targeted therapy of glioblastoma. J. Natl. Cancer Inst. *108*. https://doi.org/10.1093/jnci/djv375.

18. Burger, M.C., Zhang, C., Harter, P.N., Romanski, A., Strassheimer, F., Senft, C., Tonn, T., Steinbach, J.P., and Wels, W.S. (2019). CAR-engineered NK cells for the treatment of glioblastoma: Turning innate effectors into precision tools for cancer immunotherapy. Front. Immunol. *10*, 2683. https://doi.org/10.3389/fimmu.2019.02683.

19. Burger, M.C., Forster, M.T., Romanski, A., Straßheimer, F., Macas, J., Zeiner, P.S., Steidl, E., Herkt, S., Weber, K.J., Schupp, J., et al. (2023). Intracranial injection of natural killer cells engineered with a HER2-targeted chimeric antigen receptor in patients with recurrent glioblastoma. Neuro. Oncol. *25*, 2058–2071. https://doi.org/10.1093/neuonc/noad087.

20. Eitler, J., Wotschel, N., Miller, N., Boissel, L., Klingemann, H.G., Wels, W., and Tonn, T. (2021). Inability of granule polarization by NK cells defines tumor resistance and can be overcome by CAR or ADCC mediated targeting. J. Immunother. Cancer *9*, e001334. https://doi.org/10.1136/jitc-2020-001334.

21. Eitler, J., Rackwitz, W., Wotschel, N., Gudipati, V., Murali Shankar, N., Sidorenkova, A., Huppa, J.B., Ortiz-Montero, P., Opitz, C., Künzel, S.R., et al. (2024). CAR-mediated targeting of NK cells overcomes tumor immune escape caused by ICAM-1 downregulation. J. Immunother. Cancer *12*, e008155. https://doi.org/10.1136/jitc-2023-008155.

22. Clevers, H. (2016). Modeling development and disease with organoids. Cell *165*, 1586–1597. https://doi.org/10.1016/j.cell.2016.05.082.

23. Sachs, N., de Ligt, J., Kopper, O., Gogola, E., Bounova, G., Weeber, F., Balgobind, A. V., Wind, K., Gracanin, A., Begthel, H., et al. (2018). A living biobank of breast cancer organoids captures disease heterogeneity. Cell *172*, 373–386.e10. https://doi.org/10.1016/j.cell.2017.11.010.

24. Schnalzger, T.E., de Groot, M.H., Zhang, C., Mosa, M.H., Michels, B.E., Röder, J., Darvishi, T., Wels, W.S., and Farin, H.F. (2019). 3D model for CAR-mediated

cytotoxicity using patient-derived colorectal cancer organoids. EMBO J. *38*, e100928. https://doi.org/10.15252/embj.2018100928.

25. Rosigkeit, S., Kruchem, M., Thies, D., Kreft, A., Eichler, E., Boegel, S., Jansky, S., Siegl, D., Kaps, L., Pickert, G., et al. (2021). Definitive evidence for Club cells as progenitors for mutant Kras/Trp53-deficient lung cancer. Int. J. Cancer *149*, 1670–1682. https://doi.org/10.1002/ijc.33756.

26. Visvader, J.E., and Stingl, J. (2014). Mammary stem cells and the differentiation hierarchy: current status and perspectives. Genes Dev. *28*, 1143–1158. https://doi.org/10.1101/gad.242511.114.

27. Mariotti, L.G., Pirovano, G., Savage, K.I., Ghita, M., Ottolenghi, A., Prise, K.M., and Schettino, G. (2013). Use of the γ-H2AX assay to investigate DNA repair dynamics following multiple radiation exposures. PLoS One *8*, e79541. https://doi.org/10.1371/journal.pone.0079541.

28. Zhang, C., Röder, J., Scherer, A., Bodden, M., Pfeifer Serrahima, J., Bhatti, A., Waldmann, A., Müller, N., Oberoi, P., and Wels, W.S. (2021). Bispecific antibody-mediated redirection of NKG2D-CAR natural killer cells facilitates dual targeting and enhances antitumor activity. J. Immunother. Cancer *9*, e002980. https://doi.org/10.1136/jitc-2021-002980.

29. Wu, H.T., Zhong, H.T., Li, G.W., Shen, J.X., Ye, Q.Q., Zhang, M.L., and Liu, J. (2020). Oncogenic functions of the EMT-related transcription factor ZEB1 in breast cancer. J. Transl. Med. *18*, 51. https://doi.org/10.1186/s12967-020-02240-z.

30. Varchetta, S., Gibelli, N., Oliviero, B., Nardini, E., Gennari, R., Gatti, G., Silva, L.S., Villani, L., Tagliabue, E., Ménard, S., et al. (2007). Elements related to heterogeneity of antibody-dependent cell cytotoxicity in patients under trastuzumab therapy for primary operable breast cancer overexpressing Her2. Cancer Res. *67*, 11991–11999. https://doi.org/10.1158/0008-5472.Can-07-2068.

31. Mikaelian, I., Hovick, M., Silva, K.A., Burzenski, L.M., Shultz, L.D., Ackert-Bicknell, C.L., Cox, G.A., and Sundberg, J.P. (2006). Expression of terminal differentiation proteins defines stages of mouse mammary gland development. Vet. Pathol. *43*, 36–49. https://doi.org/10.1354/vp.43-1-36.

32. Sahu, S., Albaugh, M.E., Martin, B.K., Patel, N.L., Riffle, L., Mackem, S., Kalen, J.D., and Sharan, S.K. (2022). Growth factor dependency in mammary organoids regulates ductal morphogenesis during organ regeneration. Sci. Rep. *12*, 7200. https://doi.org/10.1038/s41598-022-11224-6.

33. Shackleton, M., Vaillant, F., Simpson, K.J., Stingl, J., Smyth, G.K., Asselin-Labat, M. L., Wu, L., Lindeman, G.J., and Visvader, J.E. (2006). Generation of a functional mammary gland from a single stem cell. Nature *439*, 84–88. https://doi.org/10.1038/nature04372.

34. Visvader, J.E. (2009). Keeping abreast of the mammary epithelial hierarchy and breast tumorigenesis. Genes Dev. *23*, 2563–2577. https://doi.org/10.1101/gad.1849509.

35. Sleeman, K.E., Kendrick, H., Robertson, D., Isacke, C.M., Ashworth, A., and Smalley, M.J. (2007). Dissociation of estrogen receptor expression and in vivo stem cell activity in the mammary gland. J. Cell Biol. *176*, 19–26. https://doi.org/10.1083/jcb.200604065.

36. Yang, X., Wang, H., and Jiao, B. (2017). Mammary gland stem cells and their application in breast cancer. Oncotarget *8*, 10675–10691. https://doi.org/10.18632/oncotarget.12893.

37. Yarden, Y., and Sliwkowski, M.X. (2001). Untangling the ErbB signalling network. Nat. Rev. Mol. Cell Biol. *2*, 127–137. https://doi.org/10.1038/35052073.

38. Hynes, N.E., and Lane, H.A. (2005). ERBB receptors and cancer: the complexity of targeted inhibitors. Nat. Rev. Cancer *5*, 341–354. https://doi.org/10.1038/nrc1609.

39. Marvalim, C., Datta, A., and Lee, S.C. (2023). Role of p53 in breast cancer progression: An insight into p53 targeted therapy. Theranostics *13*, 1421–1442. https://doi.org/10.7150/thno.81847.

40. Fischer, E.G. (2020). Nuclear morphology and the biology of cancer cells. Acta Cytol. *64*, 511–519. https://doi.org/10.1159/000508780.

41. Parekh, A., Das, S., Parida, S., Das, C.K., Dutta, D., Mallick, S.K., Wu, P.H., Kumar, B.N.P., Bharti, R., Dey, G., et al. (2018). Multi-nucleated cells use ROS to induce breast cancer chemo-resistance in vitro and in vivo. Oncogene *37*, 4546–4561. https://doi.org/10.1038/s41388-018-0272-6.

42. Cancer Genome Atlas Network (2012). Comprehensive molecular portraits of human breast tumours. Nature *490*, 61–70. https://doi.org/10.1038/nature11412.

43. Heim, C., Moser, L.M., Kreyenberg, H., Bonig, H.B., Tonn, T., Wels, W.S., Gradhand, E., Ullrich, E., Meister, M.T., Koerkamp, M.G., et al. (2023). ErbB2 (HER2)-CAR-NK-92 cells for enhanced immunotherapy of metastatic fusion-driven alveolar rhabdomyosarcoma. Front. Immunol. *14*, 1228894. https://doi.org/10.3389/fimmu.2023.1228894.

44. Mark, C., Czerwinski, T., Roessner, S., Mainka, A., Hörsch, F., Heublein, L., Winterl, A., Sanokowski, S., Richter, S., Bauer, N., et al. (2020). Cryopreservation impairs 3-D migration and cytotoxicity of natural killer cells. Nat. Commun. *11*, 5224. https://doi.org/10.1038/s41467-020-19094-0.

45. Fitzgerald, A.A., Li, E., and Weiner, L.M. (2020). 3D culture systems for exploring cancer immunology. Cancers (Basel) *13*, 56. https://doi.org/10.3390/cancers13010056.

46. Imamura, Y., Mukohara, T., Shimono, Y., Funakoshi, Y., Chayahara, N., Toyoda, M., Kiyota, N., Takao, S., Kono, S., Nakatsura, T., and Minami, H. (2015). Comparison of 2D- and 3D-culture models as drug-testing platforms in breast cancer. Oncol. Rep. *33*, 1837–1843. https://doi.org/10.3892/or.2015.3767.

47. Karlsson, H., Fryknäs, M., Larsson, R., and Nygren, P. (2012). Loss of cancer drug activity in colon cancer HCT-116 cells during spheroid formation in a new 3-D spheroid cell culture system. Exp. Cell Res. *318*, 1577–1585. https://doi.org/10.1016/j.yexcr.2012.03.026.

48. Platonova, S., Cherfils-Vicini, J., Damotte, D., Crozet, L., Vieillard, V., Validire, P., André, P., Dieu-Nosjean, M.C., Alifano, M., Régnard, J.F., et al. (2011). Profound coordinated alterations of intratumoral NK cell phenotype and function in lung carcinoma. Cancer Res. *71*, 5412–5422. https://doi.org/10.1158/0008-5472.Can-10-4179.

49. Ichise, H., Tsukamoto, S., Hirashima, T., Konishi, Y., Oki, C., Tsukiji, S., Iwano, S., Miyawaki, A., Sumiyama, K., Terai, K., and Matsuda, M. (2022). Functional visualization of NK cell-mediated killing of metastatic single tumor cells. Elife *11*, e76269. https://doi.org/10.7554/eLife.76269.

50. Ran, G.H., Lin, Y.Q., Tian, L., Zhang, T., Yan, D.M., Yu, J.H., and Deng, Y.C. (2022). Natural killer cell homing and trafficking in tissues and tumors: from biology to application. Signal Transduct. Target. Ther. *7*, 205. https://doi.org/10.1038/s41392-022-01058-z.

51. Liu, Z., Meng, X., Tang, X., Zou, W., and He, Y. (2023). Intratumoral tertiary lymphoid structures promote patient survival and immunotherapy response in head neck squamous cell carcinoma. Cancer Immunol. Immunother. *72*, 1505–1521. https://doi.org/10.1007/s00262-022-03310-5.

52. Sinha, D., Saha, P., Samanta, A., and Bishayee, A. (2020). Emerging concepts of hybrid epithelial-to-mesenchymal transition in cancer progression. Biomolecules *10*, 1561. https://doi.org/10.3390/biom10111561.

53. Bornes, L., Belthier, G., and van Rheenen, J. (2021). Epithelial-to-mesenchymal transition in the light of plasticity and hybrid E/M states. J. Clin. Med. *10*, 2403. https://doi.org/10.3390/jcm10112403.

54. Verstappe, J., and Berx, G. (2023). A role for partial epithelial-to-mesenchymal transition in enabling stemness in homeostasis and cancer. Semin. Cancer Biol. *90*, 15–28. https://doi.org/10.1016/j.semcancer.2023.02.001.

55. Brabletz, S., Schuhwerk, H., Brabletz, T., and Stemmler, M.P. (2021). Dynamic EMT: a multi-tool for tumor progression. EMBO J. *40*, e108647. https://doi.org/10.15252/embj.2021108647.

56. Terry, S., Savagner, P., Ortiz-Cuaran, S., Mahjoubi, L., Saintigny, P., Thiery, J.P., and Chouaib, S. (2017). New insights into the role of EMT in tumor immune escape. Mol. Oncol. *11*, 824–846. https://doi.org/10.1002/1878-0261.12093.

57. Mullins, R.D.Z., Pal, A., Barrett, T.F., Heft Neal, M.E., and Puram, S.V. (2022). Epithelial-mesenchymal plasticity in tumor immune evasion. Cancer Res. *82*, 2329–2343. https://doi.org/10.1158/0008-5472.Can-21-4370.

58. Gong, J.H., Maki, G., and Klingemann, H.G. (1994). Characterization of a human cell line (NK-92) with phenotypical and functional characteristics of activated natural killer cells. Leukemia *8*, 652–658.

59. Demaison, C., Parsley, K., Brouns, G., Scherr, M., Battmer, K., Kinnon, C., Grez, M., and Thrasher, A.J. (2002). High-level transduction and gene expression in hematopoietic repopulating cells using a human immunodeficiency virus type 1-based

lentiviral vector containing an internal spleen focus forming virus promoter. Hum. Gene Ther. *13*, 803–813. https://doi.org/10.1089/10430340252898984.

60. Zufferey, R., Nagy, D., Mandel, R.J., Naldini, L., and Trono, D. (1997). Multiply attenuated lentiviral vector achieves efficient gene delivery in vivo. Nat. Biotechnol. *15*, 871–875. https://doi.org/10.1038/nbt0997-871.

61. Morita, S., Kojima, T., and Kitamura, T. (2000). Plat-E: an efficient and stable system for transient packaging of retroviruses. Gene Ther. *7*, 1063–1066. https://doi.org/10.1038/sj.gt.3301206.

62. Jonkers, J., Meuwissen, R., van der Gulden, H., Peterse, H., van der Valk, M., and Berns, A. (2001). Synergistic tumor suppressor activity of BRCA2 and p53 in a conditional mouse model for breast cancer. Nat. Genet. *29*, 418–425. https://doi.org/10.1038/ng747.

63. Bankhead, P., Loughrey, M.B., Fernández, J.A., Dombrowski, Y., McArt, D.G., Dunne, P.D., McQuaid, S., Gray, R.T., Murray, L.J., Coleman, H.G., et al. (2017). QuPath: Open source software for digital pathology image analysis. Sci. Rep. *7*, 16878. https://doi.org/10.1038/s41598-017-17204-5.

YMTHE, Volume *33*

Supplemental Information

ErbB2/HER2-targeted CAR-NK cells eliminate breast cancer cells in an organoid model that recapitulates tumor progression

Jasmin Röder, Tijna Alekseeva, Anne Kiefer, Ines Kühnel, Maren Prüfer, Congcong Zhang, Malena Bodden, Sebastian Rosigkeit, Anja Waldmann, Torsten Tonn, Ernesto Bockamp, Stefan Stein, and Winfried S. Wels

Supplemental Methods

ImageJ Macro to determine organoid area based on brightfield images

Language: ImageJ Macro

```
// Select the brightfield image
selectWindow("Brightfield");
// Apply Gaussian Blur to smooth the image
run("Gaussian Blur...", "sigma=1");
// Set scale in microns
run("Set Scale...", "distance=17.0 known=100 unit=micron");
// Set automatic threshold and convert to mask
//setThreshold(137, 255);
setOption("BlackBackground", true);
run("Convert to Mask");
// Apply morphological operations to clean up the mask
run("Close-");
run("Erode");
run("Watershed");
// Analyze particles (organoid area)
run("Analyze Particles...", "size=9000-400000 show=[Count Masks] display exclude clear include overlay
add composite");
```

Image-based analysis of organoid area. The macro processes a brightfield image by applying a Gaussian blur to smoothen the image and reduce noise. The image scale is set to microns using a known distance for calibration. A threshold segments the organoids, converting the binarized image into a mask. Morphological operations, including erosion and watershed, refine the mask and separate closely connected organoids. Particle analysis measures organoid sizes within a specified range, and results are displayed.

ImageJ Macro to determine nucleus area based on Hoechst staining

Language: ImageJ Macro

```
// Select the Hoechst image
selectWindow("Hoechst");
// Set scale in microns
selectWindow("Hoechst");
run("Set Scale...", "distance=312 known=100 unit=micron");
// Enhance contrast
run("Enhance Contrast", "saturated=0.35");
// Set threshold for nuclei
setAutoThreshold("Huang dark");
run("Convert to Mask");
// Apply morphological operations to clean up the mask
run("Erode");
run("Watershed");
// Analyze particles (nuclei area)
run("Analyze Particles...", "size=20-20000 display exclude clear include overlay add");
```

Image-based analysis of nucleus area. The macro processes a Hoechst-stained image to measure nucleus size. The image scale is set to microns using a known distance for calibration. The contrast of the image is enhanced, followed by automatic thresholding to segment the nuclei, which converts the image into a mask. Morphological operations, such as erosion and watershed, are applied to refine the mask and separate closely connected nuclei. Particle measures the size of the nuclei within a specified size range, and the results are displayed.

ImageJ Macro to quantify mean fluorescent intensity of p53 staining

Language: ImageJ Macro

```
// Select the image (Composite-p53-Hoechst)
selectWindow("Composite");
// Split the channels of the composite image
run("Split Channels");
selectWindow("C1-Composite");
rename("p53");
selectWindow("C2-Composite");
rename("Hoechst");
// Adjust brightness and contrast
selectWindow("Hoechst");
setMinAndMax(95, 316);
run("8-bit");
selectWindow("p53");
setMinAndMax(152, 407);
run("8-bit");
// Apply pseudo flat field correction to Hoechst
selectWindow("Hoechst");
run("Pseudo flat field correction", "blurring=1");
// Create background mask from Hoechst channel
selectWindow("Hoechst");
run("Duplicate...", "title=Hoechst_background");
selectWindow("Hoechst_background");
run("Median...", "radius=1");
setOption("BlackBackground", true);
run("Convert to Mask");
run("Watershed");
// Analyze particles
run("Analyze Particles...", "size=80-Infinity circularity=0.50-1.00 exclude overlay add composite");
selectWindow("p53");
roiManager("Measure");
```

Image-based analysis of p53 expression. The macro is designed to process a composite image containing Hoechst and p53 channels to measure p53 expression within nuclei. The composite image is split into two separate channels, with the first channel designed for p53 and the second for Hoechst. Brightness and contrast adjustments are made for both channels, and the Hoechst channel undergoes a pseudo flat field correction to enhance uniformity. A duplicate of the Hoechst channel is created and processed with a median filter to generate a background mask. The background mask is then converted to a binary mask and refined using a watershed segmentation. Particle analysis is conducted on the mask to identify and select nuclei based on predefined size and circularity parameters. These regions of interest (ROIs) are then used to measure p53 signal intensity within the p53 channel (mean fluorescence intensity; MFI), and the results are displayed.

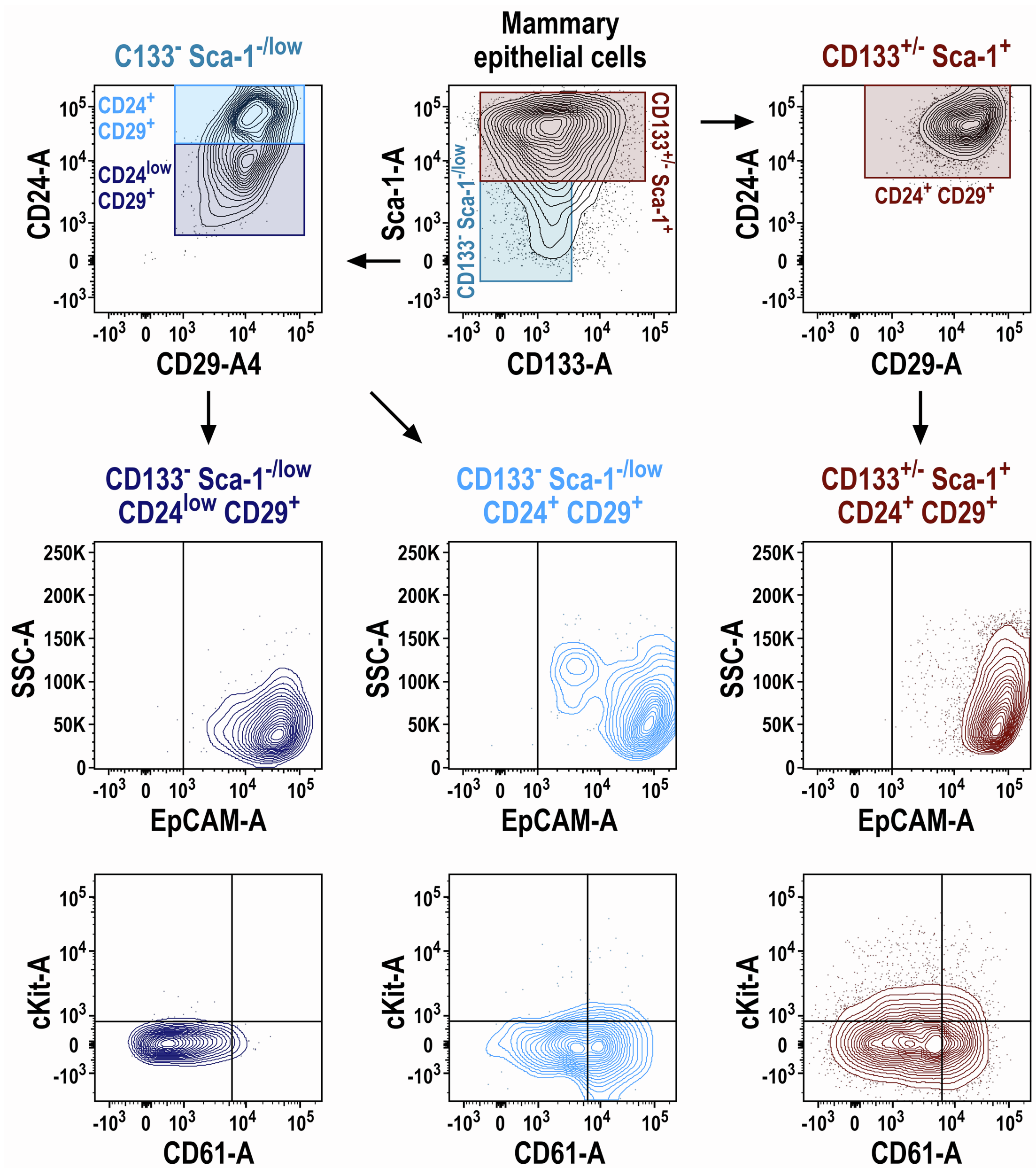

Figure S1. Cellular heterogeneity of murine mammary epithelial cells in 3D culture
Flow cytometric analysis of dissociated organoids stained with antibodies against surface proteins CD24, CD29, CD61, CD133, c-Kit, Sca-1 and EpCAM. Basal lineages and mammary stem cells ($CD24^{low}CD29^{+}CD133^{-}EpCAM^{+}Sca\text{-}1^{-/low}$) are depicted in dark blue, while luminal progenitors ($CD24^{+}CD29^{+}CD133^{+/-}EpCAM^{high}Sca\text{-}1^{+}CD61^{+}cKit^{+/-}$) and fully differentiated ductal and alveolar cells ($CD24^{+}CD29^{+}CD133^{+/-}EpCAM^{high}Sca\text{-}1^{+}CD61^{-}cKit^{-/low}$) are indicated in brown. Light blue represents a mixture of epithelial lineages.

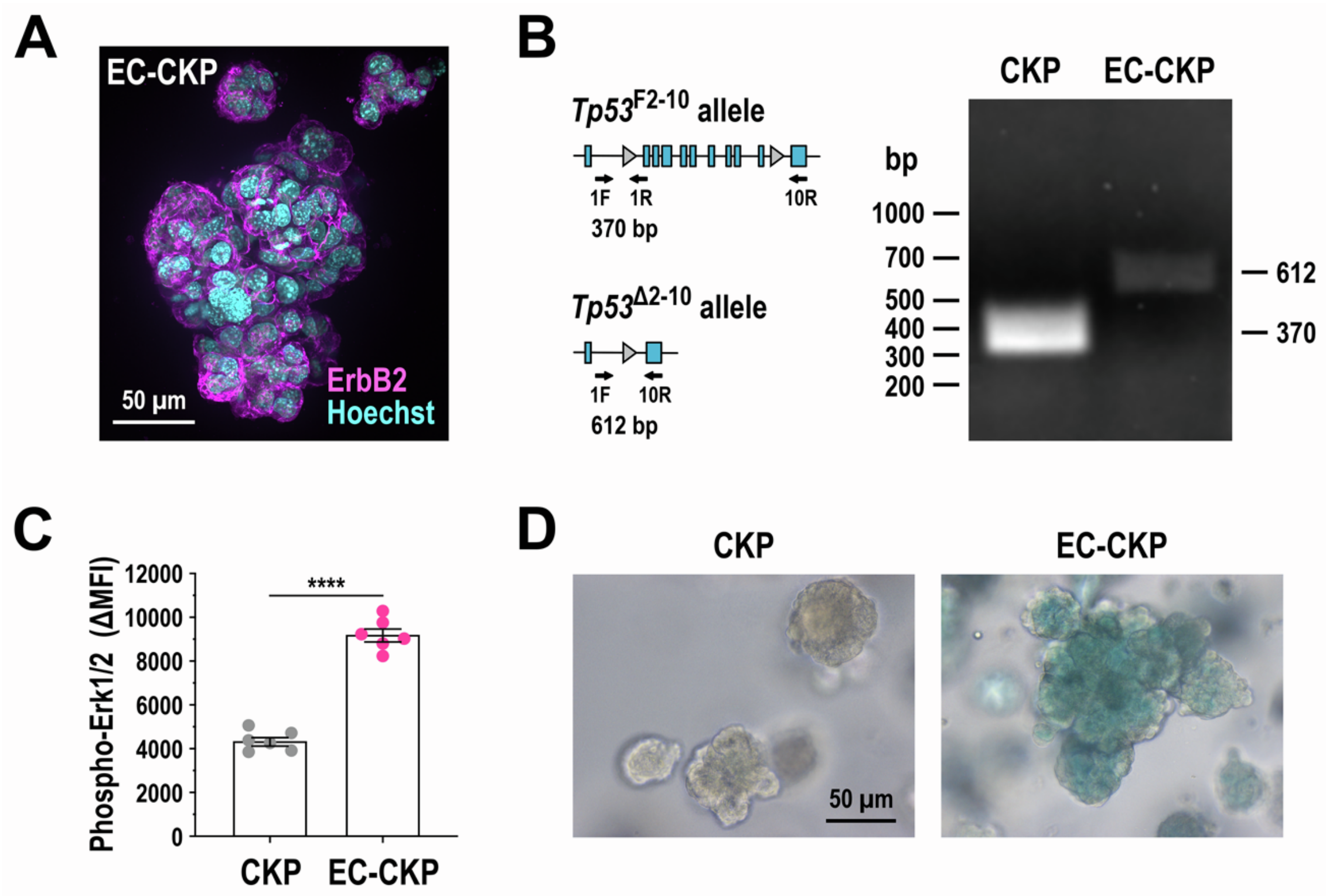

Figure S2. Genetic driver mutations in EC-CKP cells.
(A) Analysis of EC-CKP organoids by immunofluorescence microscopy. Organoids were fixed in Matrigel, stained with an ErbB2-specific antibody (magenta) and Hoechst dye (blue), and analyzed via confocal microscopy. Shown is a maximum intensity projection processed from images of a representative organoid. Scale bar: 50 µm. (B) Schematic representation of the PCR analysis detecting the *Tp53*$^{F2\text{-}10}$ allele with LoxP sequences (gray arrowheads) in introns 1 and 10, and the recombined *Tp53*$^{\Delta 2\text{-}10}$ allele lacking exons 2 to 10 (blue rectangles) after Cre-mediated recombination (left panel). PCR was performed with primers 1F, 1R, and 10R (black arrows), generating specific products of 370 base pairs (bp) in the case of unmodified CKP organoids, and 612 base pairs in the case of ErbB2- and Cre-expressing EC-CKP organoids carrying the recombined *Tp53*$^{\Delta 2\text{-}10}$ allele (right panel). (C) Flow cytometric analysis of phosphorylated Erk1/2. Organoids were dissociated, fixed, permeabilized, and then subjected to staining with a phospho-Erk1/2-specific antibody followed by species-specific fluorochrome-conjugated secondary antibody. Mean values ± SEM of delta mean fluorescence intensities (ΔMFI) from six independent experiments are shown. ****, $p < 0.0001$. (D) Enzymatic activity of the β-Geo reporter linked to Kras^{G12V} expression detected by X-Gal staining of CKP and EC-CKP organoids in Matrigel. Scale bar: 50 µm.

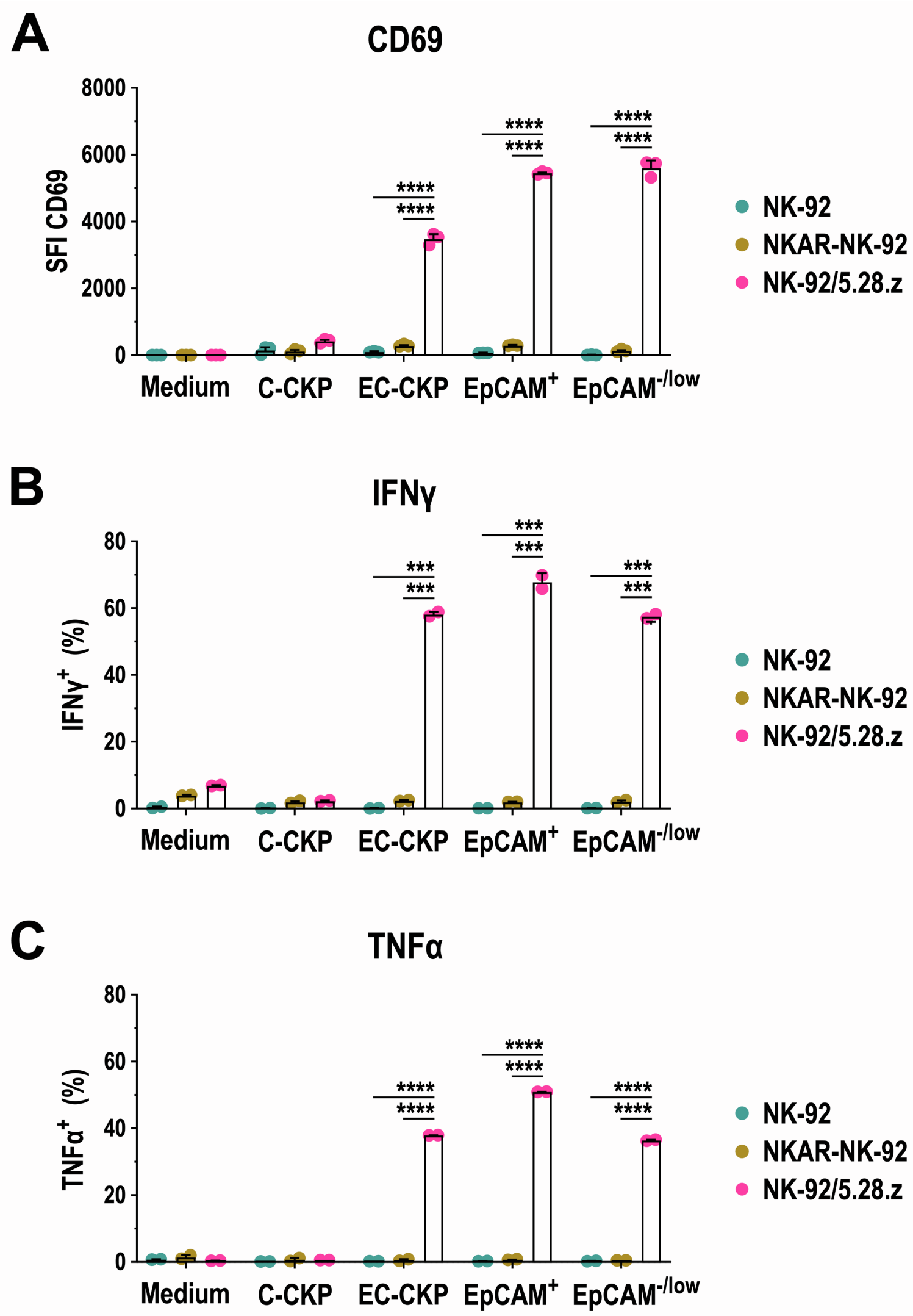

Figure S3. Activation of ErbB2-specific NK-92/5.28.z cells by *in vivo* passaged organoids
(A) Expression of the activation marker CD69 by NK-92/5.28.z cells upon co-culture with dissociated C-CKP or EC-CKP organoids from continuous *in vitro* culture, or $EpCAM^{+}$ and $EpCAM^{-/low}$ EC-CKP tumor cells from the second *in vivo* passage was analyzed after 4 hours by flow cytometry using a CD69-specific antibody (SFI, specific fluorescence intensity). Parental NK-92 cells and NKAR-NK-92 cells expressing a CAR specific for human NKG2D ligands were included for comparison. NK cells incubated in the absence of target cells (medium) served as control. (B, C) Cytokine expression by NK-92/5.28.z cells after co-incubation with target cells for 4 hours as indicated in (A) was analyzed by intracellular staining. Percentages of IFNγ- (B) and TNFα-positive cells (C) were determined by flow cytometry. Mean values ± SD of n=3 (A) or n=2 (B, C) biological replicates are shown. ****, $p < 0.0001$; ***, $p < 0.001$.

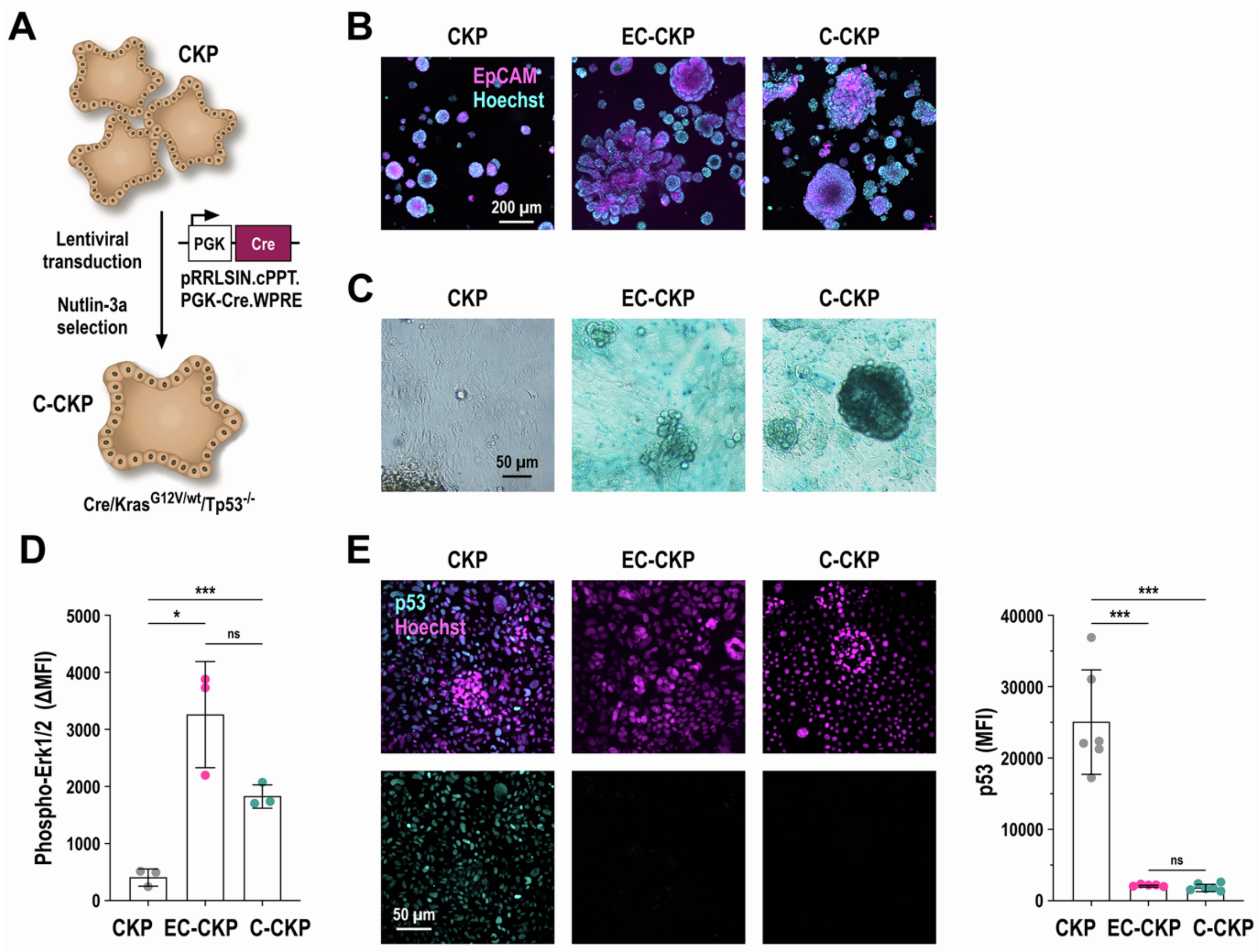

Figure S4. Establishment of mammary organoids with neoplastic driver mutations that lack ErbB2 expression

(A) For conditional activation of oncogenic KRas^{G12V} and knockout of p53 without concomitant ErbB2 expression, mammary organoids from the CKP mouse were modified via lentiviral transduction to express Cre recombinase, and selected for p53 knockout cells in medium containing 10 µM Nutlin-3a (C-CKP). (B) Morphology of newly formed CKP, EC-CKP and C-CKP organoids in Matrigel was assessed by immunofluorescence microscopy five days after seeding of fully dissociated organoid cells. Cells were stained with anti-EpCAM antibody (magenta) and counterstained with Hoechst dye (cyan). Z-sections were obtained by confocal microscopy and are presented as maximum intensity projections. Scale bar: 200 µm. (C) X-Gal staining of 2D cultures derived from parental CKP and genetically modified EC-CKP and C-CKP organoids. Scale bar: 50 µm. (D) Flow cytometric analysis of phosphorylated Erk1/2 proteins in CKP, EC-CKP and C-CKP organoids. Single-cell suspensions were fixed, permeabilized, and stained with phospho-ERK1/2-specific antibody, followed by fluorochrome-labeled secondary antibody. Mean values ± SD of delta mean fluorescence intensities (ΔMFI) from three independent experiments are shown. ***, $p < 0.001$; *, $p < 0.05$; ns, $p \geq 0.05$. (E) Expression of p53 by mammary organoids was analyzed by immunofluorescence microscopy. Dissociated CKP, EC-CKP and C-CKP organoids were cultured as 2D monolayers, fixed, permeabilized, and then stained with a p53-specific antibody, followed by fluorochrome-conjugated secondary antibody (cyan) and counterstaining with Hoechst dye (magenta). Shown are representative maximum intensity projections of z-sections (left panels). Scale bar: 50 µm. Mean fluorescence intensities (MFI) were determined using ImageJ by processing Hoechst channel images to create a binary mask, allowing quantification of p53 signals in the nuclei (right panel). Mean values ± SEM determined from at least five independent microscopic images of each cell type are shown. ***, $p < 0.001$; ns, $p \geq 0.05$.

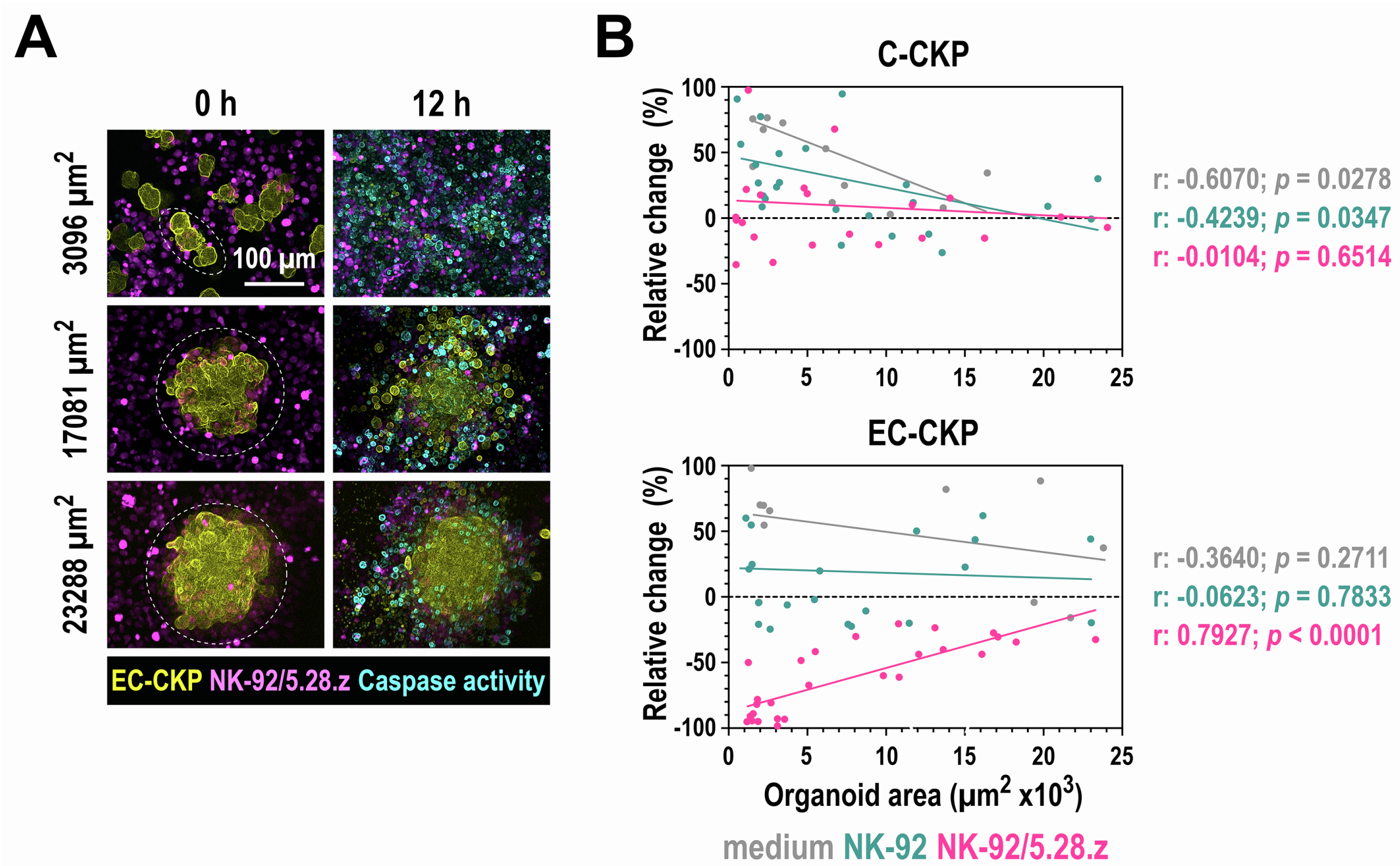

Figure S5. Size-dependent lysis of mammary organoids by CAR-NK cells

(A) For microscopic analysis of target cell killing, ErbB2-expressing EC-CKP organoids carrying a Luc2-sfGFP marker gene construct (yellow) were co-cultured with NK-92/5.28.z cells (magenta) for 12 hours in the presence of a fluorescent caspase-3/7 substrate (cyan). Shown are processed maximum intensity projections (MIP) of representative organoids of small, medium and large size (3096, 17081 and 23288 μm^2) imaged at 0 and 12 hours. Scale bar: 100 µm. (B) For quantitative analysis of size-dependent lysis of ErbB2-negative C-CKP and ErbB2-expressing EC-CKP organoids by NK-92/5.28.z and parental NK-92 cells, MIP-based hyperstacks were generated from microscopic images, and MFI values of the sfGFP signal at the beginning (0 h) and end (12 h) of the experiment were determined using ImageJ software. The relative change in signal intensity after 12 hours was calculated for each organoid and plotted against organoid size (μm^2). Organoids kept without NK cells served as control (medium). Shown are scatter plots of individual organoids from five independent experiments with corresponding linear regressions and correlation coefficients (r).

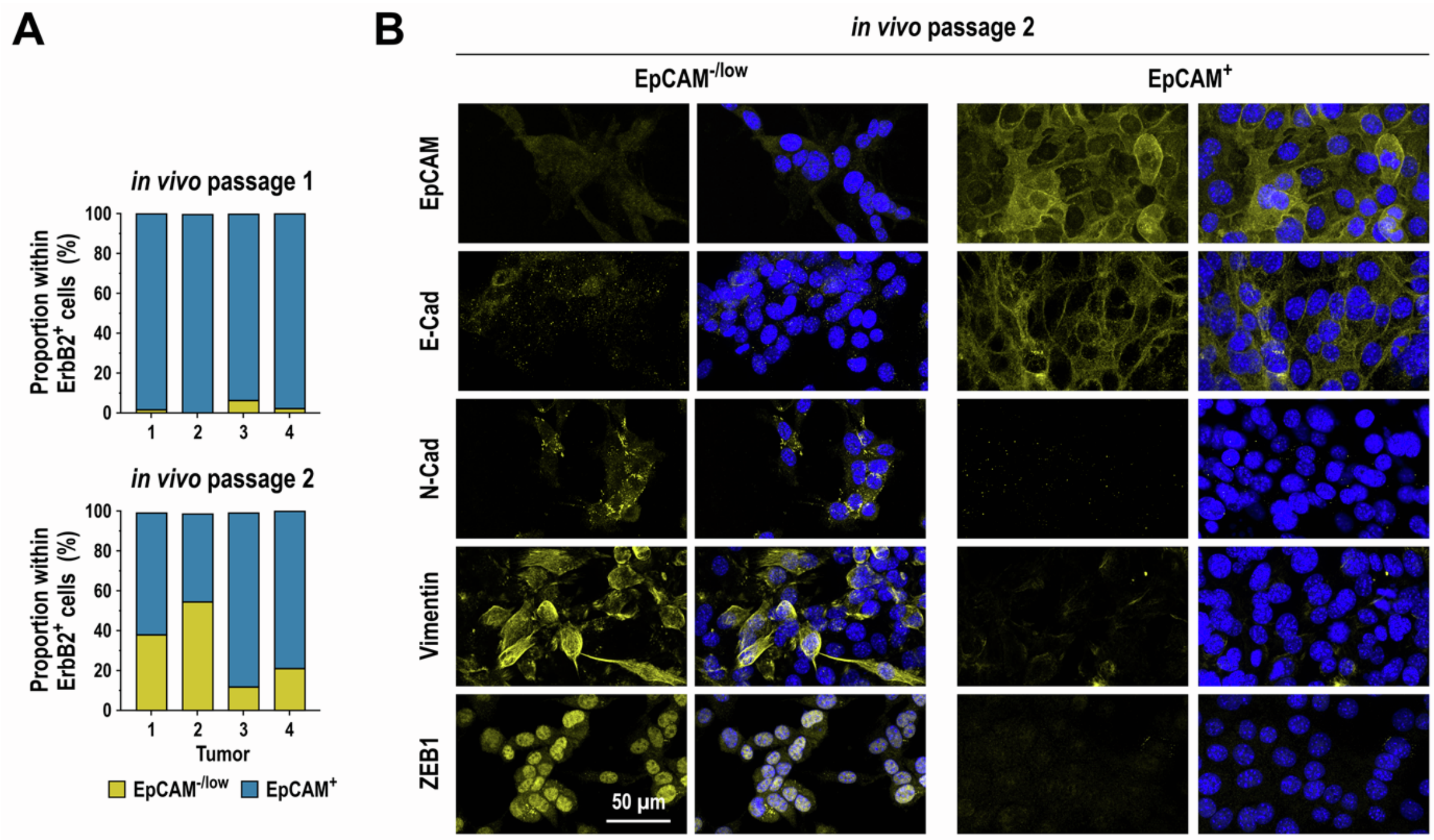

Figure S6. EMT phenotype of *in vivo* passaged organoids

(A) Single-cell suspensions of EC-CKP tumor organoid cultures after *in vivo* passage 1 (top) or 2 (bottom) were analyzed for EpCAM surface expression using flow cytometry. Data are shown as percentage of $EpCAM^{-/low}$ and $EpCAM^{+}$ cells within the ErbB2-positive population for organoids derived from four individual tumors. (B) Sorted $EpCAM^{-/low}$ and $EpCAM^{+}$ cells from *in vivo* passage 2 growing as 2D cultures were fixed, permeabilized and stained with antibodies specific for EpCAM, E-Cadherin (E-Cad), N-Cadherin (N-Cad), Vimentin and ZEB1, followed by species-specific fluorochrome-conjugated secondary antibodies. Counterstaining was performed using Hoechst dye. Shown are representative maximum intensity projections depicting the EMT markers (yellow; left panels) and merged images including the Hoechst staining (blue; right panels). Scale bar: 50 µm.

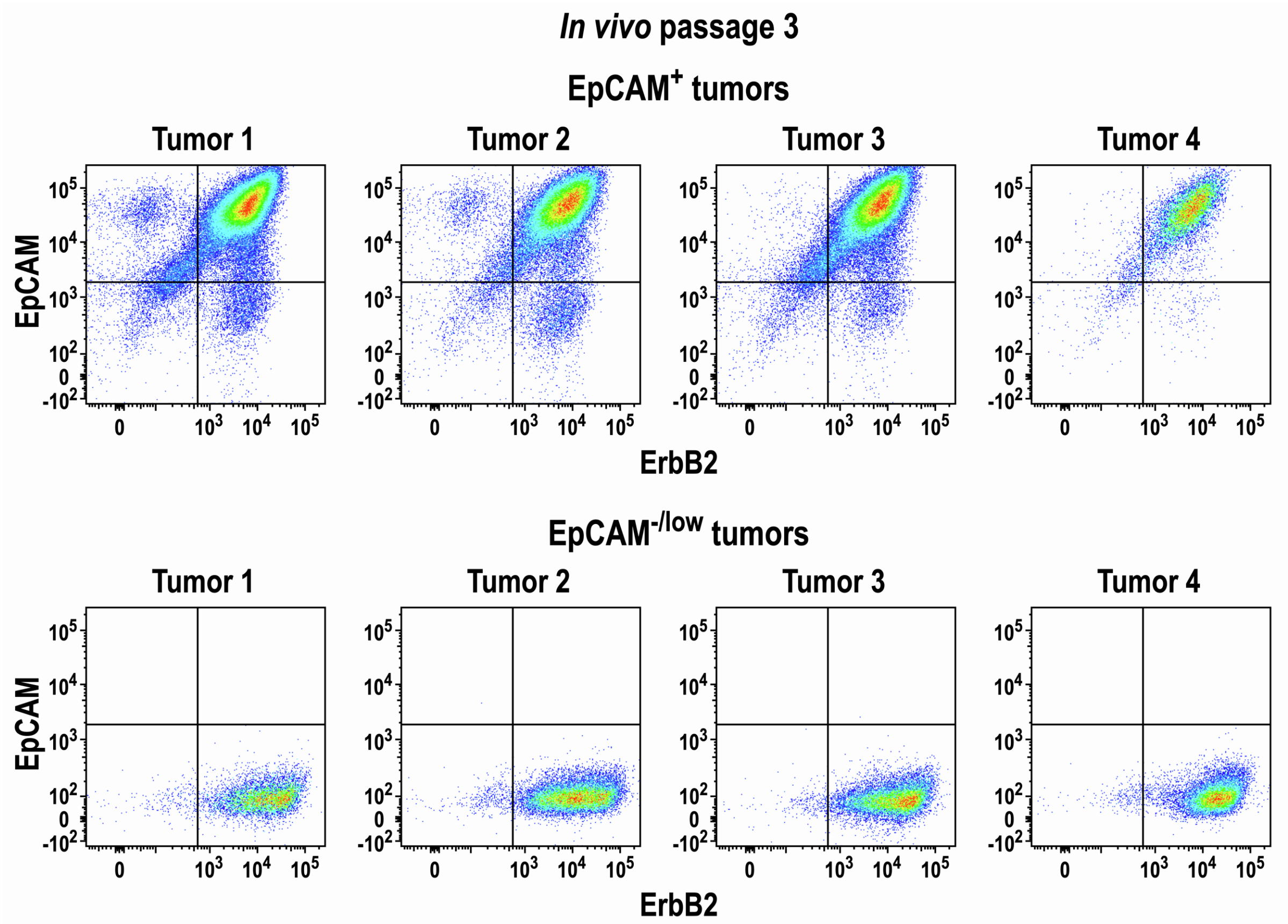

Figure S7. ErbB2 expression in tumors from *in vivo* passage 3
Subcutaneous tumors from the experiment shown in Figure 5E were excised, dissociated into single-cell suspensions and expanded for two weeks before flow cytometric analysis with EpCAM-specific and ErbB2-specific antibodies. Data for tumors from four individual animals of each group are shown.

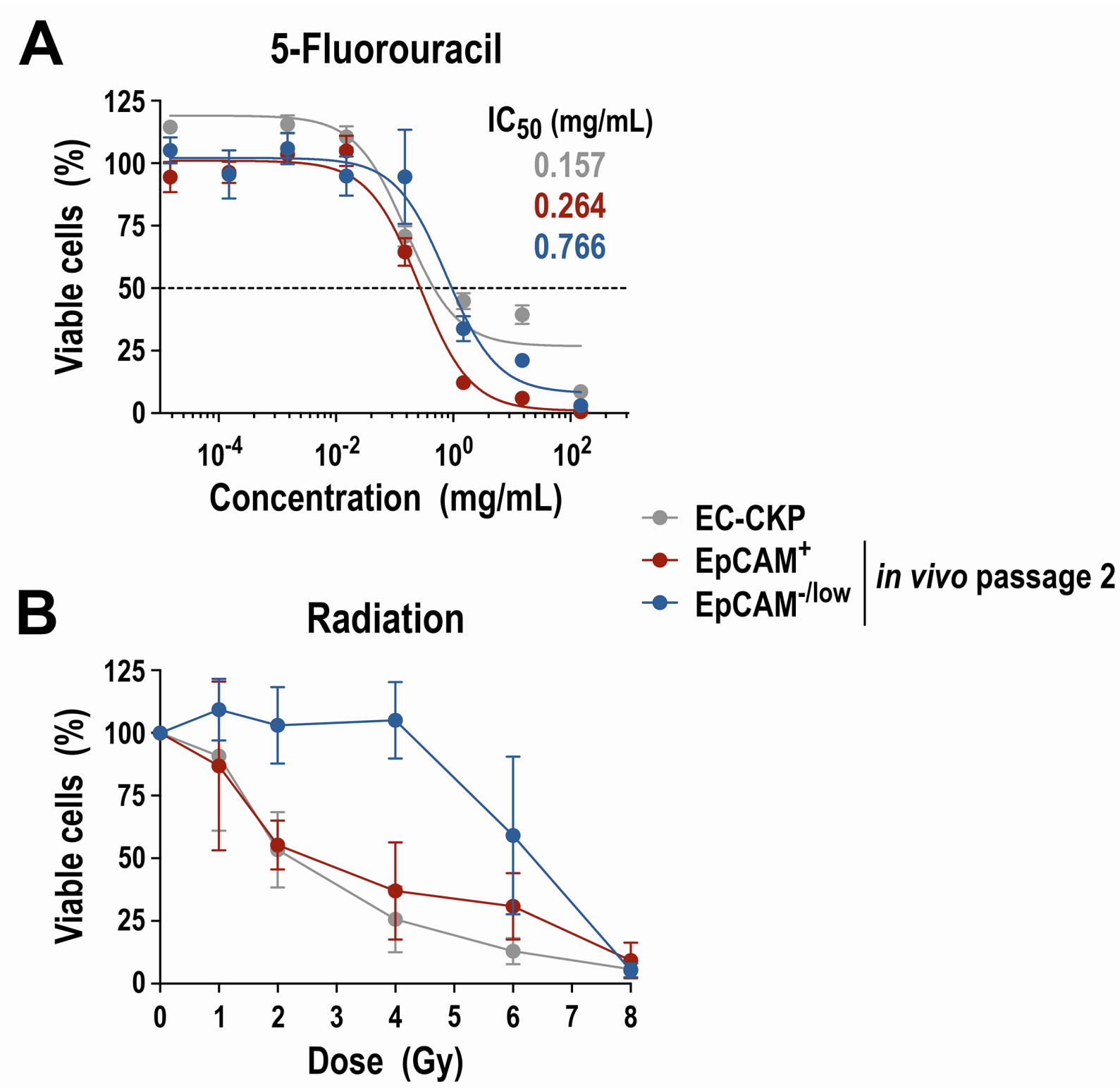

Figure S8. Sensitivity of *in vivo* passaged organoids to chemotherapy and radiation

(A) Single-cell suspensions of *in vitro* cultured EC-CKP organoids (gray) and *in vivo* passaged EpCAM^{+} (red) and EpCAM$^{-/low}$ cells (blue) tumor organoids were seeded in 96-well plates, and increasing concentrations of 5-fluorouracil were added the following day. After 5 days, cell viability was evaluated using a resazurin-based assay. For this, medium was replaced with resazurin solution (44 µM in fresh culture medium), and fluorescence was measured after 1-2 hours of incubation with a SpectraMax iD3 microplate reader (excitation: 530 nm, emission: 590 nm, integration time: 100 ms). The proportion of viable cells was calculated based on fluorescence values obtained from untreated and staurosporine-treated controls (5 µM). Mean values ± SD of n=3 technical replicates are shown. IC_{50} values were determined from dose response curves calculated using GraphPad Prism. (B) Single-cell suspensions of organoids as described in (A) were irradiated at doses of 2, 4, 6 and 8 Gy using a BioBeam 2000 GM irradiation system, followed by seeding in 12-well plates. After 7 days of culture, the proportion of viable cells compared to untreated controls was determined using a crystal violet assay. Cells were fixed with 4% paraformaldehyde and stained with of 0.5% crystal violet in 20% methanol. Cell-bound crystal violet was solubilized for 30 minutes using Sorenson's buffer (0.1 M sodium citrate, 50% EtOH, pH 4.2), and absorbance at 540 nm was measured using a SpectraMax iD3 microplate reader. Mean values ± SD of n=4 technical replicates are shown.

Supplemental Movies

Movies S1 and S2. Cytotoxic activity of CAR-NK cells against neoplastic mammary organoids
ErbB2-negative C-CKP (Movie S1) and ErbB2-expressing EC-CKP organoids (Movie S2) carrying a Luc2-sfGFP marker gene construct (yellow) were co-cultured with NK-92/5.28.z cells (magenta) for 12 hours in the presence of a fluorescent caspase-3/7 substrate (cyan). Microscopic images were acquired every 15 minutes, maximum intensity projections were derived, and assembled into MPEG-4 movies. The time stamp indicates minutes:seconds. Individual images taken at 0, 180, 360, 540 and 705 minutes of the experiment are shown in Figure 4C.
